# Intrastriatal administration of coenzyme Q10 enhances neuroprotection in a Parkinson’s disease rat model

**DOI:** 10.1038/s41598-020-66493-w

**Published:** 2020-06-12

**Authors:** Hyung Woo Park, Chun Gwon Park, Min Park, Seung Ho Lee, Hye Ran Park, Jaesung Lim, Sun Ha Paek, Young Bin Choy

**Affiliations:** 1 Department of Neurosurgery, Seoul National University College of Medicine, Seoul, 03080 Republic of Korea; 2 Cancer Research Institute, Seoul National University College of Medicine, Seoul, 03080 Republic of Korea; 3 Ischemic/Hypoxic Disease Institute, Seoul National University College of Medicine, Seoul, 03080 Republic of Korea; 4 Department of Biomedical Engineering, SKKU Institute for Convergence, Sungkyunkwan University (SKKU), Suwon, 16419 Republic of Korea; 5 Biomedical Institute for Convergence at SKKU (BICS), Sungkyunkwan University, 2066 Seobu-ro, Jangan-gu, Suwon, 16419 Republic of Korea; 6 Interdisciplinary Program in Bioengineering, College of Engineering, Seoul National University, Seoul, 08826 Republic of Korea; 7 Institute of Medical & Biological Engineering, Medical Research Center, Seoul National University, Seoul, 03080 Republic of Korea; 8 Department of Biomedical Engineering, Seoul National University College of Medicine, Seoul, 03080 Republic of Korea

**Keywords:** Drug delivery, Parkinson's disease, Preclinical research

## Abstract

Parkinson’s disease is a neurodegenerative disorder, and no treatment has been yet established to prevent disease progression. Coenzyme Q10, an antioxidant, has been considered a promising neuroprotective agent; however, conventional oral administration provides limited efficacy due to its very low bioavailability. In this study, we hypothesised that continuous, intrastriatal administration of a low dose of Coenzyme Q10 could effectively prevent dopaminergic neuron degeneration. To this end, a Parkinson’s disease rat model induced by 6-hydroxydopamine was established, and the treatment was applied a week before the full establishment of this disease model. Behavioural tests showed a dramatically decreased number of asymmetric rotations in the intrastriatal Coenzyme Q10 group compared with the no treatment group. Rats with intrastriatal Coenzyme Q10 exposure also exhibited a larger number of dopaminergic neurons, higher expression of neurogenetic and angiogenetic factors, and less inflammation, and the effects were more prominent than those of orally administered Coenzyme Q10, although the dose of intrastriatal Coenzyme Q10 was 17,000-times lower than that of orally-administered Coenzyme Q10. Therefore, continuous, intrastriatal delivery of Coenzyme Q10, especially when combined with implantable devices for convection-enhanced delivery or deep brain stimulation, can be an effective strategy to prevent neurodegeneration in Parkinson’s disease.

## Introduction

Parkinson’s disease is the second most common neurodegenerative disorder^1^, which affects approximately 7 million people globally. With increased life expectancy, the disease is expected to have an even greater impact in the future^2^. Parkinson’s disease is a progressive disorder caused by the death of dopaminergic cells in the substantia nigra and a consequent reduction of dopamine in the striatum^1^. Since the progressive loss of dopamine in the basal ganglia causes the motor symptoms of Parkinson’s disease, therapy in clinical settings is predominantly focused on the exogenous supply of dopamine with the prodrug, levodopa (L-dopa), or dopamine agonists, to offer symptomatic relief. However, such therapeutic approaches are effective mainly in the early stage of the disease^3,4^. Moreover, prolonged use of L-dopa or dopamine agonists has been reported to become ineffective at improving motor symptoms and often provokes severe side effects, such as joint stiffness, dyskinesia, somnolence, oedema, and hallucinations^4^.

Consequently, many attempts have been made to identify potent novel drugs (i.e. motor- or non-motor symptomatic agents) or innovative drug-delivery methods for treating Parkinson’s disease^5,6^. Nevertheless, there is a paucity of effective treatments to address the progression of Parkinson’s disease, i.e. the prevention of the continual loss of dopaminergic cells. Approximately 50% of dopaminergic neurons are impaired at the time of first diagnosis, and an additional 45% are progressively impaired within the subsequent decade^7,8^. The slow progression of Parkinson’s disease during this later period creates the opportunity for disease interventions by suppressing the loss of nigral cells or promoting their recovery.

Nigral cell death is caused by mitochondrial dysfunction, which is triggered by abnormal energy metabolism and enhanced cellular oxidative stress^9,10^. In this regard, there is substantial interest in exploring the use of antioxidants such as coenzyme Q10 (CoQ10) as potential therapies for Parkinson’s disease^11,12^. CoQ10 is a lipophilic substance involved in various essential cellular processes and is a potent antioxidant in mitochondrial and lipid membranes^13^. However, oral supplementation with CoQ10 is often limited by its low bioavailability at the site of interest, such as the striatum. Systemic absorption of orally administered CoQ10 is very low due to its relatively large molecular weight (864.34 g/mol) and very low aqueous solubility (≤53.2 µg/mL)^14^. In addition, CoQ10 in the blood stream rarely reaches the striatum owing to the blood-brain barrier^15,16^. Despite the widespread attempts to enhance CoQ10 bioavailability^17,18^, Parkinson’s disease patients still require daily administrations of a very high dose of CoQ10 (300–3000 mg/day) for weeks to months^19,20^. Therefore, patients are more likely to have difficulty maintaining the regimen. As such, therapeutic efficacy cannot be easily guaranteed^21^.

Localised intrastriatal delivery may be a potential means to improve the bioavailability of CoQ10. Unlike oral routes, this direct exposure can achieve a therapeutically effective concentration of CoQ10 even with a significantly reduced dose. Thus, CoQ10 can be continuously infused into the brain, as already established with convection-enhanced delivery (CED) in clinical settings^22,23^. However, studies on strategies for intrastratial delivery of CoQ10 are scarce. Therefore, we aimed to investigate the therapeutic efficacy of CoQ10 delivered locally into the striatum, which was tested in 6-hydroxydopamine (6-OHDA)-induced rat models of Parkinson’s disease. To assess the effects on the prevention of disease progression, animals were treated with CoQ10 3 weeks after 6-OHDA treatment, i.e. a week before the time generally accepted for the full establishment of Parkinson’s disease^24^. For intrastriatal delivery, CoQ10 was infused at a constant rate through a catheter inserted into the striatum using an Alzet osmotic pump (Model 2ML4; Durect, CA, USA). The progression of nigral cell death was compared with that in animals treated with a larger dose of orally administered CoQ10.

## Results

### CoQ10 treatment

To examine the effects of locally delivered CoQ10 and its doses in preventing Parkinson’s disease progression, we utilised an Alzet osmotic pump (2ML4; reservoir volume: 2000 µL; flow rate: 2.5 µL/h; Durect, CA, USA) as a tool for continuous, intrastriatal infusion of CoQ10^25^. The details of the pump used herein are described in Supplementary Fig. S1. To vary the dose, CoQ10 solutions of two different concentrations (25 and 40 µg/mL) were employed to produce the Alzet-low CoQ10 and Alzet-high CoQ10 pumps, respectively. An Alzet pump filled with pH 7.4 phosphate-buffered saline (PBS) without CoQ10 was also prepared to produce the Alzet-PBS as a control.

In this work, we prepared five distinct animal groups, depending on treatment: (1) no treatment (animals without treatment, n = 3), (2) oral CoQ10 (animals treated with oral administration of CoQ10, n = 3), (3) Alzet-PBS (animals treated with an Alzet pump filled with PBS only, n = 3), (4) Alzet-low CoQ10 (animals treated with an Alzet pump filled with a 25 μg/mL CoQ10 solution, n = 3), and (5) Alzet-high CoQ10 (animals treated with an Alzet pump filled with a 40 μg/mL CoQ10 solution, n = 5).

**Figure 1:**
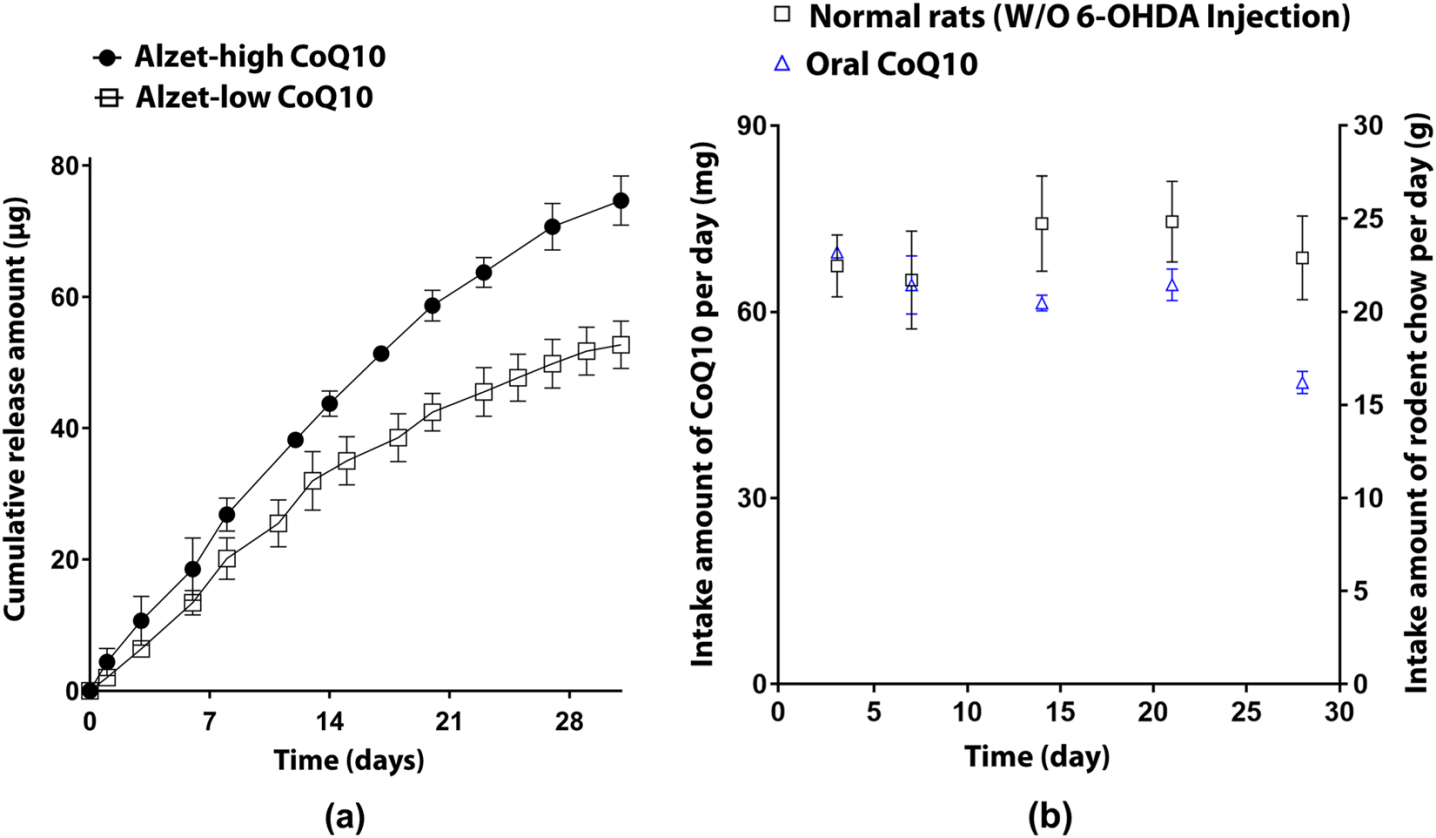
CoQ10 delivery profiles via intrastriatal and oral routes. (**a**) *In vitro* drug release profiles of CoQ10 from the Alzet pump. Each pump was fully immersed and shaken at 100 rpm in 20 mL of PBS (pH 7.4) at 37 °C (JeoiTech, Seoul, Korea), where one end of the catheter was connected to the pump and the other end was linked to the collection tube. At scheduled time points for 35 days, the solution in the collection tube was fully extracted and assayed spectrophotometrically at 270 nm to measure the amount of infused CoQ10. The experiments were performed in triplicate for Alzet-low CoQ10 and Alzet-high CoQ10, respectively. (**b**) Profiles of the amount of oral intake of CoQ10 calibrated from that of rodent chow. For this, 1 g of rodent chow was mixed with 3 mg of CoQ10, enabling the administration of 60 mg/day CoQ10 per rat according to the average weight and amount of intake per day of rats (approximately 300 g and 20 g, respectively). The CoQ10-mixed chow was sterilised and stored away from light exposure until use^75^.

With the pumps loaded with CoQ10, we evaluated the *in vitro* drug release profile in pH 7.4 PBS. As illustrated in Fig. 1a, CoQ10 release patterns were fairly linear for 28 days for both Alzet-low CoQ10 and Alzet-high CoQ10 pumps (R^2^ > 0.97). The pumps infuse dCoQ10 intrastriatally at an average rate of 1.8 and 2.6 µg per day for the Alzet-low CoQ10 and Alzet-high CoQ10 groups, respectively^26^, resulting in total release amounts of 50.72 and 71.64 µg for 4 weeks, respectively.

For the oral CoQ10 group, rodent chow mixed with CoQ10 (3 mg CoQ10 per g chow) was fed freely to animals treated with 6-OHDA. As shown in Fig. 1b, the oral CoQ10 group consumed >20 g of chow every day for 3 weeks, indicating oral administration of > 60 mg CoQ10 per day (i.e.>200 mg/kg) during this period. Subsequently, chow intake decreased slightly compared to that of normal animals without 6-OHDA injection, possibly due to the progression of neurodegeneration^27,28^. However, the amount of intake was maintained at >15 g per day, indicating a daily CoQ10 dose of > 45 mg per day (i.e. >150 mg/kg per day). Thus, the CoQ10 dose of the oral CoQ10 group was at least 25,000- and 17,000-times higher than that of the Alzet-low CoQ10 and Alzet-high CoQ10 groups, respectively (Fig. 1a).

### Behavioural analysis

**Figure 2:**
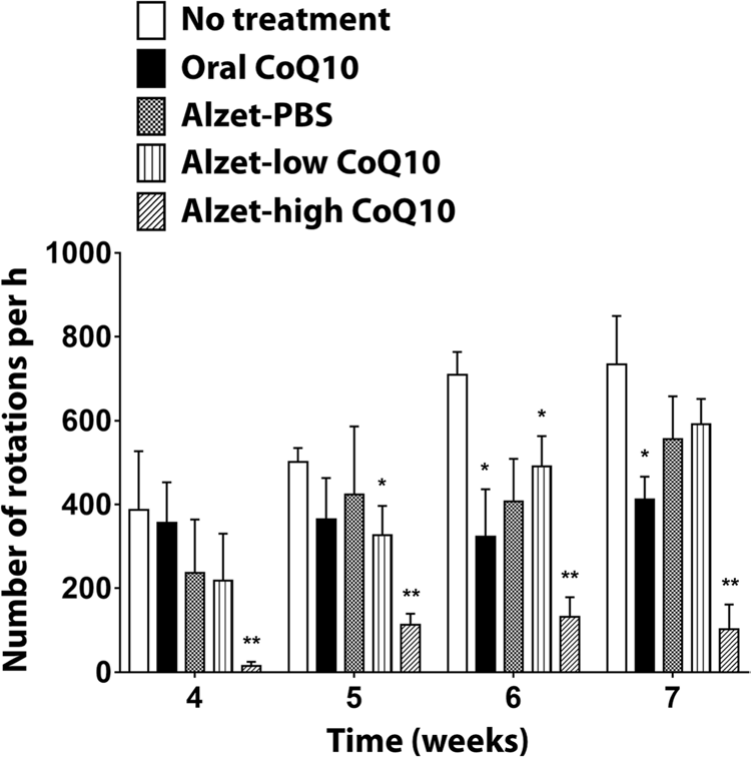
Apomorphine-induced rotational behaviours of animals in a Parkinson’s disease model. ^*^Significantly different from the no treatment group (p < 0.05). ^**^Significantly different from all other groups (p < 0.05).

To evaluate the degree of neurodegeneration, rotation tests were performed on Parkinson’s disease rats, as illustrated in Fig. 2 (see also Supplementary Video S1)^29^. Upon administration of apomorphine, rats began to rotate asymmetrically due to neurodegeneration induced by 6-OHDA, where an increase in the number of rotations represents more severely damaged neurons^30^.

In the absence of treatment (i.e. no treatment group), the number of rotations gradually increased until 7 weeks, suggesting continuous degeneration of dopaminergic cells in 6-OHDA-treated animals^31^. For the oral CoQ10 group, there was no apparent increase in the number of rotations during the testing periods at 4–7 weeks, implying an effect of orally administered CoQ10 on the prevention of neurodegeneration progression^32,33^. Thus, the number of rotations was significantly lower than that of the no treatment group at 6 and 7 weeks (p < 0.05). This delayed efficacy could be attributed to the delayed increase in CoQ10 concentration in the brain that is often observed with oral administration of CoQ10^34^.

When CoQ10 was delivered intrastriatally at a dose of 1.8 µg per day (i.e. the Alzet-low CoQ10 group), the number of rotations was lower than that of the no treatment group; however, a significant difference was observed only at 5 and 6 weeks (p < 0.05). Moreover, the number of rotations was not significantly different between the Alzet-PBS and Alzet-low CoQ10 groups throughout the entire testing period (p > 0.05). The efficacy of the low dose of CoQ10 was hardly distinguishable from that observed with insertion of a cannula near the striatum^35–37^.

Notably, when CoQ10 was delivered into the brain at an increased dose of 2.6 µg per day, there was an apparent decrease in the number of rotations: the number of rotations for the Alzet-high CoQ10 group was significantly lower than that for all other groups throughout the entire testing period (p < 0.05). It should be noted that this prominent efficacy was observed with a dose that was at least 17,000 times lower than that of the oral CoQ10 group, implying very high bioavailability of intrastriatally delivered CoQ10.

### Evaluation of dopaminergic neurons

**Figure 3:**
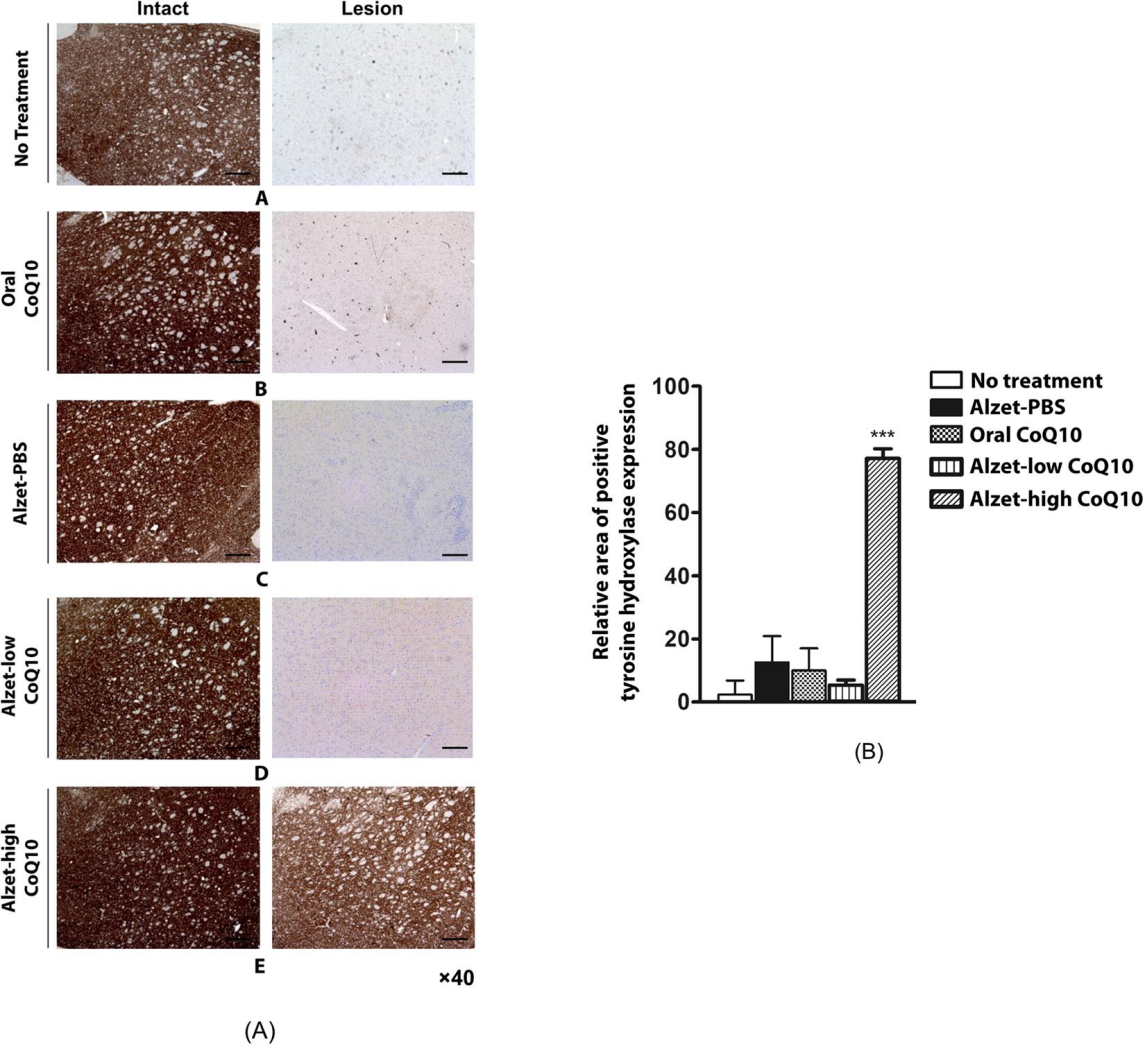
Tyrosine hydroxylase expression in the intact (left) and lesioned (right) striatum. (A) Images were obtained of the (**a**) no treatment group, (**b**) oral CoQ10 group, (**c**) Alzet-PBS group, (**d**) Alzet-low CoQ10 group, and (**e**) Alzet-high CoQ10 group. The scale bars are 100 µm. (B) Quantitative analysis results obtained from all images (Supplementary Fig. S5). ***Significantly different to all other groups (p < 0.001).

To further examine the efficacy of CoQ10 in the prevention of neurodegeneration, we assessed the expression of tyrosine hydroxylase in the striatum with biopsied brain tissues at the experimental endpoint. Tyrosine hydroxylase expression in the striatum is reported to be low due to reduced dopamine observed in most Parkinson’s disease patients^38–40^. As shown in Fig. 3, strong staining was observed in all groups in the intact left hemisphere, which was not treated with 6-OHDA, indicating normal dopamine levels. However, tyrosine hydroxylase expression was rarely observable in the lesioned right hemisphere for the no treatment group, indicating that most dopaminergic cells were destroyed at 7 weeks after 6-OHDA treatment.

For the oral CoQ10 group, tyrosine hydroxylase expression was observable, suggesting that oral administration of CoQ10 at a dose of > 45 mg per day (i.e. >150 mg/kg per day) partly elicited a neuroprotective effect. The Alzet-PBS and Alzet-low CoQ10 groups exhibited scarce expression of tyrosine hydroxylase, implying that the efficacy observed for rotational behaviours originated mostly from the insertion of a cannula near the striatum in both groups (Fig. 2)^35–37^. Notably, the Alzet-high CoQ10 group exhibited the highest tyrosine hydroxylase expression in the right hemisphere treated with 6-OHDA, which showed staining almost as strong as that in the intact left hemisphere.

**Figure 4:**
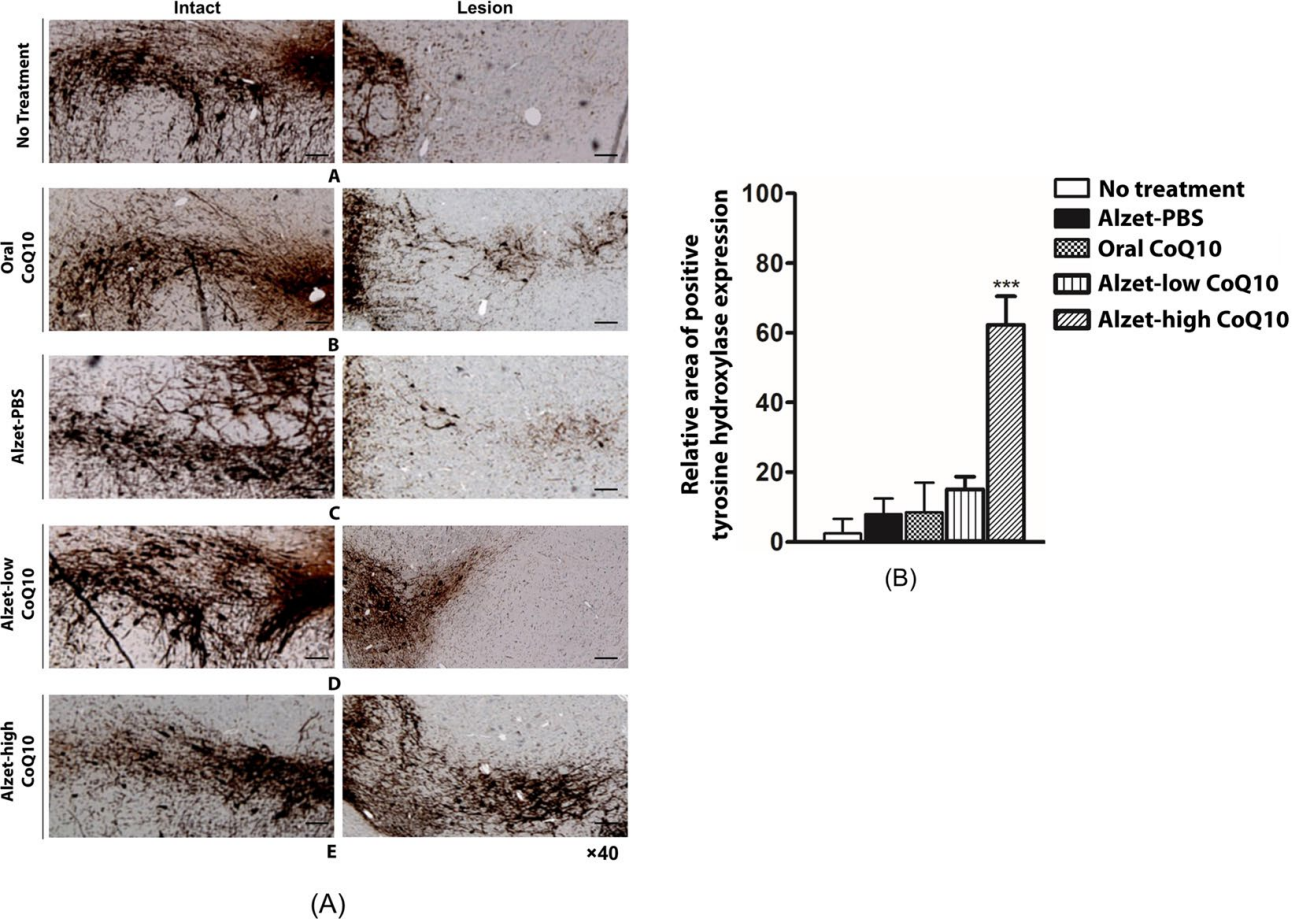
Tyrosine hydroxylase expression in the intact (left) and lesioned (right) substantia nigra. (A) Images were obtained of the (**a**) no treatment group, (**b**) oral CoQ10 group, (**c**) Alzet-PBS group, (**d**) Alzet-low CoQ10 group, and (**e**) Alzet-high CoQ10 group. The scale bars are 100 µm. (B) Quantitative analysis results obtained from all images (Supplementary Fig. S6). ***Significantly different to all other groups (p < 0.001).

We also examined the expression of tyrosine hydroxylase in the substantia nigra, where the loss of dopaminergic neurons leads to reduction of dopamine in the striatum^41^. As shown in Fig. 4, a band of tyrosine hydroxylase expression was clearly observed in the intact hemisphere for all groups; this was absent in lesioned tissue in the no treatment group. Compared to the no treatment group, the oral CoQ10 group exhibited a slightly higher expression of tyrosine hydroxylase, but the staining was not as strong as that in the intact hemisphere. Both the Alzet-PBS and Alzet-low CoQ10 groups exhibited very low expression of tyrosine hydroxylase, which was markedly lower than that observed in the oral CoQ10 group. The highest tyrosine hydroxylase expression was observed in the Alzet-high CoQ10 group, where staining was comparable to that in the intact hemisphere; notably, it was stronger than that in the oral CoQ10 group. These results indicated that intrastriatally delivered CoQ10 had a significant neuroprotective effect, which was more prominent than that of orally administered CoQ10 at a very high dose.

### Evaluation of neurogenesis, angiogenesis, and neuroinflammation

**Figure 5:**
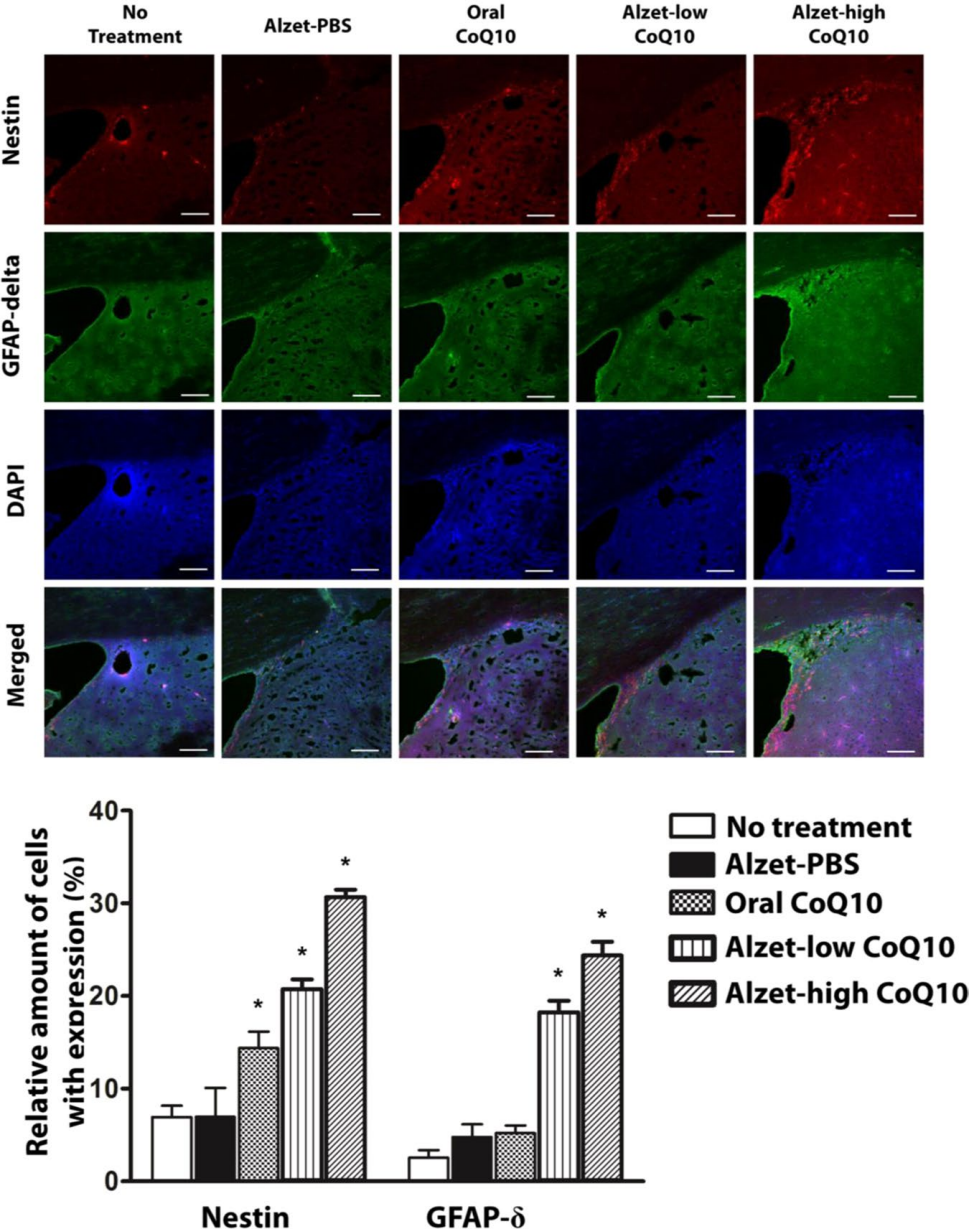
Immunofluorescence analysis of neurogenesis in the sub-ventricular zone. Nestin- and GFAP-δ-positive cells in images (top) and amounts (bottom). The scale bars are 100 µm. ^*^Significantly different to all other groups (p < 0.05).

To investigate whether intrastriatal delivery of CoQ10 was capable of inducing neurogenesis, immunofluorescence staining for nestin and glial fibrillary acidic protein (GFAP)-δ was performed^42,43^. As shown in Fig. 5, the number of cells expressing nestin and GFAP-δ was significantly higher in the Alzet-low and Alzet-high CoQ10 groups than in the no treatment group (p < 0.05), as well as in the Alzet-high CoQ10 group than in the Alzet-low group (p < 0.05). In addition, the number of cells with nestin expressions was significantly higher in the oral CoQ10 group than in the no treatment group but was statistically significantly lower in the oral CoQ10 group than in the Alzet-low CoQ10 and Alzet-high CoQ10 groups (p < 0.05). Moreover, the number of cells expressing GFAP-δ was not statistically significantly different between the oral CoQ10 group and the no treatment group. A similar number of cells with both nestin and GFAP-δ staining were observed in the Alzet-PBS group and the no treatment group.

**Figure 6:**
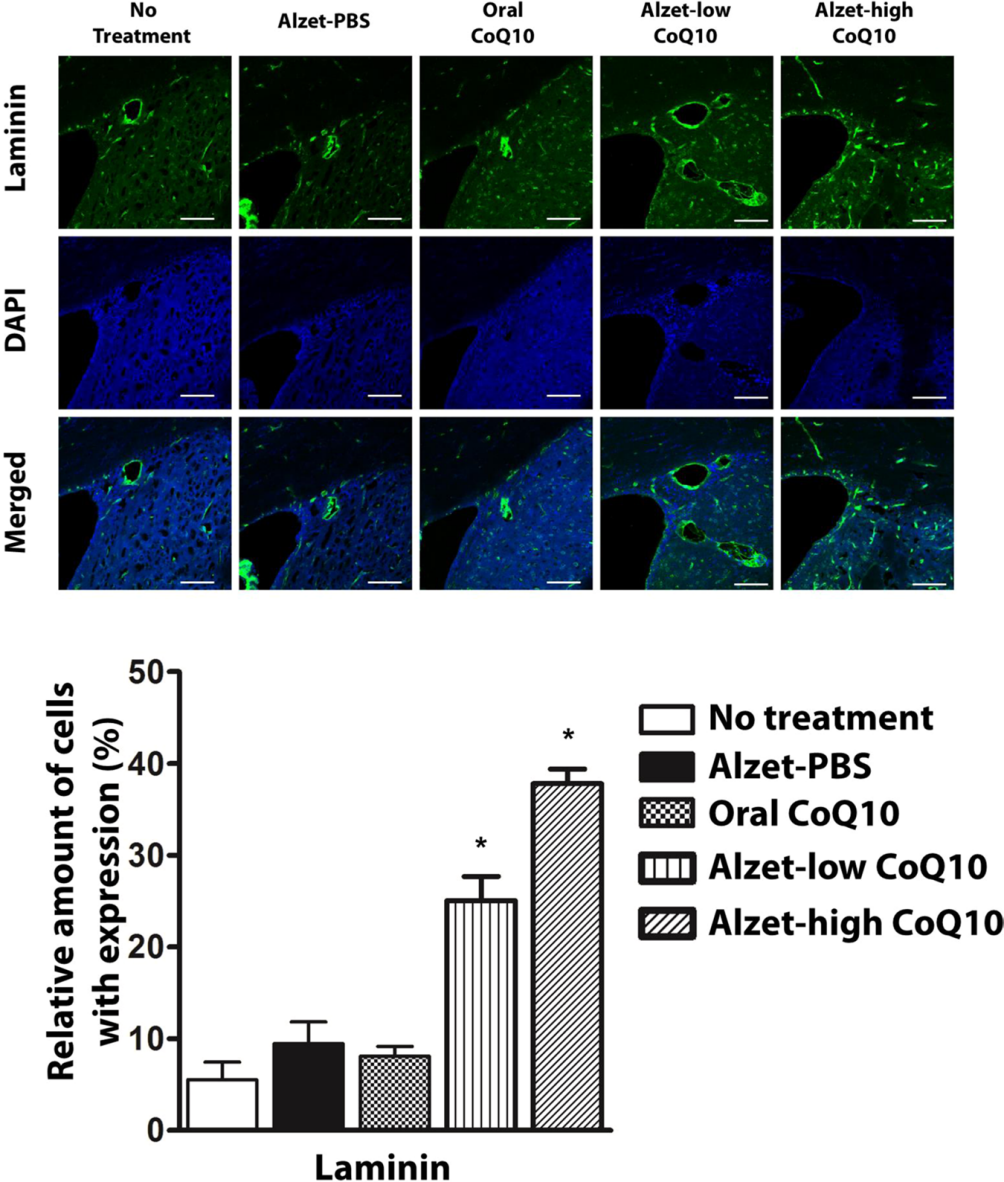
Immunofluorescence analysis of angiogenesis in the sub-ventricular zone. Laminin-positive cells in images (top) and amounts (bottom). The scale bars are 100 µm. ^*^Significantly different to all other groups (p < 0.05).

**Figure 7:**
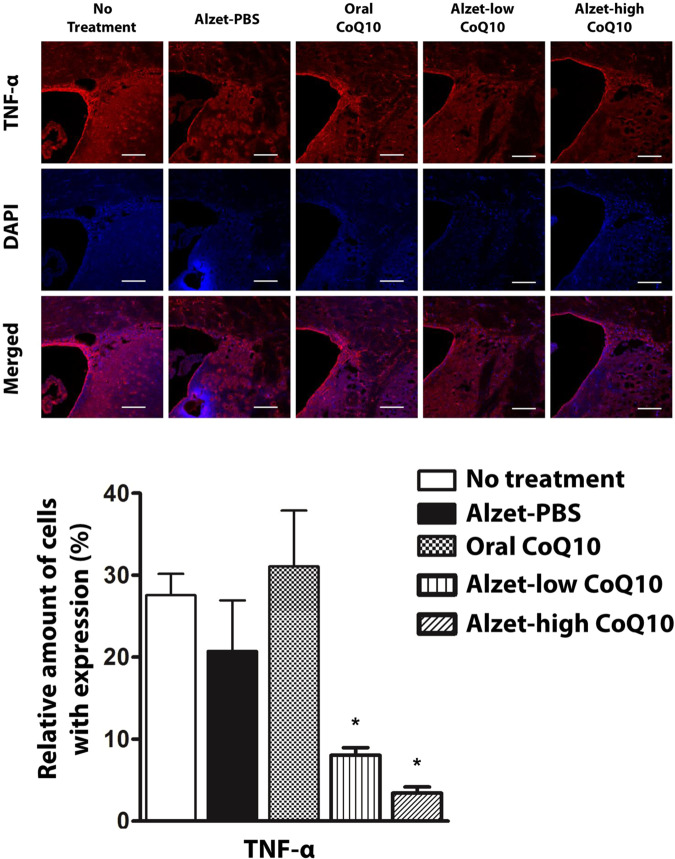
Immunofluorescence analysis of inflammation in the sub-ventricular zone. TNF-α-positive cells in images (top) and amounts (bottom). The scale bars are 100 µm. ^*^Significantly different to all other groups (p < 0.05).

Angiogenic effects of CoQ10 were observable in tissues stained with anti-laminin antibodies^26,44^. As shown in Fig. 6, the Alzet-low CoQ10 and Alzet-high CoQ10 groups exhibited significantly higher expression of laminin than did the no treatment group (p < 0.05). Between the two pump groups, laminin expression was higher in the Alzet-high CoQ10 group (p < 0.05). Compared to the no treatment group, the oral CoQ10 group did not exhibit a significant change in laminin expression. With regards to anti-inflammatory effects of CoQ10^45,46^, the expression of tumour necrosis factor (TNF)-α was decreased in both the Alzet-low CoQ10 and Alzet-high CoQ10 groups (Fig. 7), which was significantly lower than that in the no treatment group (p < 0.05). This effect was not observable in the oral CoQ10 group despite the higher dose of CoQ10 administered compared to that for groups treated with local administration of CoQ10.

## Discussion

Parkinson’s disease is the second most prevalent neurodegenerative disease. It is caused by the progressive and selective loss of dopamine-producing neurons in the substantia nigra^1^. Therefore, at the early stage of Parkinson’s disease, therapy is based mainly on supplementing dopamine by prescribing L-dopa or dopamine agonists to temporarily alleviate disease symptoms^47^. There is a paucity of treatments available in clinical settings that address the main issue of Parkinson’s disease, i.e. preventing disease progression^48^. In this regard, the neuroprotective agent CoQ10 has been actively investigated for its potential ability to suppress the progression of Parkinson’s disease^24,32,49^. CoQ10 is known to protect neuronal cells from oxidative stress and prevent the loss of the mitochondrial membrane potential^11,12^. However, due to the poor aqueous solubility of CoQ10 and its relatively high molecular weight, orally administered CoQ10 has limited systemic absorption^50^. Furthermore, the blood-brain barrier hampers the intrastriatal bioavailability of CoQ10^51,52^, and hence high doses and frequent administrations are required^53^.

To address these issues, in the present study, we proposed a therapeutic strategy of intrastriatal delivery of CoQ10 to improve its local bioavailability and prevent the progression of Parkinson’s disease. To test this approach, we employed animals 3 weeks after 6-OHDA injections, when neurodegeneration had yet to stabilise, thereby representing the early stage of Parkinson’s disease^54^. Our behavioural assessments revealed a dramatic decrease in the number of rotations when CoQ10 was intrastriatally delivered in a continuous manner compared to that of control groups (Fig. 2). In the absence of treatment, the number of rotations gradually increased in animals injected with 6-OHDA, representing progression of Parkinson’s disease in the animal model employed herein.

Our results revealed that tyrosine hydroxylase expression in the striatum and substantia nigra was higher with intrastriatal delivery of CoQ10 than it was in control animals. Notably, it was as high as that observed in the intact left hemisphere, implying substantial prevention of disease progression (Figs. 3 and 4). This could be ascribed to the fact that intrastriatal delivery of CoQ10 inhibited cell apoptosis and led to an elevation of factors facilitating neuronal growth (Fig. 5)^55^ along with enhanced angiogenesis (Fig. 6)^56,57^. The level of a pro-inflammatory cytokine, TNF-α, was also markedly reduced following local administration of CoQ10 (Fig. 7)^45,46^, which could be another influencing factor in neuroprotection as a degree of inflammation is known to be highly related to Parkinson’s disease^58,59^.

These preventive effects were still evident with the Alzet-low CoQ10 group when compared with the no treatment, Alzet-PBS and Oral CoQ10 groups. However, it should be noted that those effects were still statistically significantly lower than those of the Alzet-high CoQ10 group. This implies the presence of the dose threshold to actually influence the behavioral effects, as well as tyrosine hydroxylase expression (Figs. 2–4), which were apparent only with the Alzet-high CoQ10 in this work. However, the CoQ10 dose of intrastriatal delivery was still 17,000-times lower than that used in oral administration. Although shown to be pharmacodynamically effective, our study is limited in an aspect that the pharmacokinetics and metabolisms of CoQ10 were not fully elucidated in the local brain tissues. In addition, the study was performed with a limited number of animals representing an early stage of Parkinson’s disease. More detailed experiments including a larger number of animals, as well as those at an advanced stage of disease, would provide more information regarding to the benefits of an intrastriatal delivery strategy of CoQ10.

Although invasive, the strategy of intrastriatal CoQ10 delivery may still enable high bioavailability in the target site, which could eventually achieve therapeutic efficacy in the prevention of Parkinson’s disease progression. For conventional oral administration, a high dose of CoQ10 needs to be administered almost daily to produce a therapeutic window of CoQ10 levels in the striatum^51,52^; however, such a regimen would be difficult to follow^60^. Thus, inconsistent administration and/or drop outs are likely, leading to unsuccessful treatment of irreversible disease progression. For intrastriatal local drug administrations, various implantable systems have been studied extensively for delivery of L-dopa, dopamine, neurotrophic factors, or nerve growth factors^61^. Among them, intrastriatal implantation of a tube or electrode has already been adapted in clinical settings for convection-enhanced delivery or deep brain stimulation, respectively^16^. In this work, we were able to prepare an aqueous solution of 40 µg/mL CoQ10, which was effective in rats when continuously infused into the striatum at a volume of 60 µL per day (Figs. 1 and 2). Considering the size of the human brain^62^, the same solution is expected to be similarly effective when delivered at a relatively low volume of approximately 12 mL per day in humans. This daily infusion volume is comparable to that typically employed in convection-enhanced delivery in clinical settings (1.4–14 mL/day)^63^. Although there was a significant difference in rotational behaviour (Fig. 2), the biopsied tissues did not quite exhibit an apparent change between the groups treated with orally- and intrastriatally-delivered CoQ10 (Figs. 3–7) possibly due to the limited number of animals in each group and a relatively high dose of orally-administered CoQ10 in this study. Therefore, the strategy herein may not be comparable yet to other, more effective formulations for oral delivery, such as ubiquinol formulations^64–66^.

Our study reports, for the first time, the therapeutic efficacy of intrastriatally delivered CoQ10 to maximise its neuroprotective effects, thereby suppressing neurodegeneration to inhibit the progression of Parkinson’s disease. CoQ10 is known to elicit effects in models of other neurodegenerative diseases, such as Huntington’s disease, progressive supranuclear palsy, and Alzheimer’s disease^9^. Therefore, the intrastriatal delivery of CoQ10 described herein is a promising strategy to prevent the progression of various neurodegenerative diseases, including Parkinson’s disease.

## Methods

### Materials

Tween 80, 6-hydroxydopamine (6-OHDA), ascorbic acid (0.02%), paraformaldehyde, sucrose solutions, desipramine, and R-apomorphine hydrochloride were purchased from Sigma (MO, USA). Xylazine and Zoletil 50 were purchased from Bayer (Seoul, South Korea) and Vibac SA (Carros, France), respectively. Mouse anti-tyrosine hydroxylase (MAB5280), mouse anti-nestin (MAB353), and rabbit anti-GFAP-δ (AB9598) primary antibodies were purchased from EMD Millipore (MA, USA). Rabbit anti-laminin (L9393) and rabbit anti-TNF-α (ab66579) primary antibodies were obtained from Sigma (MO, USA) and Abcam (MA, USA), respectively. All secondary antibodies (Alexa 488-conjugated goat anti-rabbit, Alexa 594-conjugated goat anti-rabbit, and Alexa 594-conjugated goat anti-mouse IgG) were purchased from Molecular Probes (Invitrogen, CA, USA). Surgical sutures were obtained from Ethicon (4–0 Nylon; NJ, USA). Phosphate buffered saline (PBS) at pH 7.4 and 0.9% NaCl solution were obtained from Seoul National University Hospital Biomedical Research Institute (Seoul, South Korea) and Choongwae (Seoul, South Korea), respectively. Rodent chow was provided by Feedlab (Guri, South Korea). CoQ10 was a kind gift from Somang Cosmetics (Incheon, South Korea).

### CoQ10 loading in alzet osmotic pumps

To load CoQ10 in pumps, we first dissolved CoQ10 in PBS (pH 7.4) containing 2% w/v Tween 80^67^. The reservoir in the pump body was then filled with 2 mL of the resulting CoQ10 solution, which was connected to a catheter that was also filled with the same CoQ10 solution. The Alzet-low CoQ10 and Alzet-high CoQ10 pumps were filled with CoQ10 solutions of 25 and 40 µg/mL, respectively. The Alzet-PBS pump was filled with PBS (pH 7.4) containing 2% w/v Tween 80 without CoQ10. Prior to use, the pump body and catheter were primed with PBS (pH 7.4) at 37 °C for 24 h to activate the osmotic pump and remove any possible dead volume. According to the manufacturer’s specifications^26^, the infusion rate of the pump was set at 2.5 µL/h, resulting in daily CoQ10 delivery of 1.5 and 2.4 µg for the Alzet-low CoQ10 and Alzet-high CoQ10, respectively. Given the reservoir volume of 2,000 µL, the pump was expected to deliver the loaded solution for more than 4 weeks.

### *In vivo* animal evaluation

#### Animals

*In vivo* animal experiments were performed on male Sprague Dawley rats weighing 280–350 g and housed under standard laboratory conditions (12 h light/dark cycle) with food and water provided *ad libitum*. All animals were cared for in accordance with the Guide for Care and Use of Laboratory Animals of National Institutes of Health Publication (No. 85-23, revised 2011, 8^th^ edition). The study protocol was approved by the Institutional Animal Care and Use Committee at Seoul National University Hospital Biomedical Research Institute (IACUC no. 11-0114).

#### Animal model of parkinson’s disease

In this study, unilateral 6-OHDA lesioned rats were used as an animal model of Parkinson’s disease (Supplementary Fig. S2), where the lesion was made on the right hemisphere (i.e., the ipsilateral side) while the left hemisphere (i.e., the contralateral side) was left intact. For this, rats were first anaesthetised via an intraperitoneal injection of a cocktail of xylazine (5 mg/kg) and zoletil (20 mg/kg). Then, the animals were secured in a stereotaxic apparatus (David Kopf Instruments, CA, USA) and intraperitoneally injected with desipramine (25 mg/kg) to protect noradrenergic neurons prior to 6-OHDA lesioning. Subsequently, the skin was pulled back, and a burr hole was trephined in the right frontal bone using a micro hand drill. A Hamilton syringe was used to inject 12 μg of 6-OHDA dissolved in a 4 μL sterile solution of 0.9% NaCl and 0.02% ascorbic acid into the medial forebrain bundle (MFB) of the right hemisphere at a flow rate of 0.5 μL/min. The stereotaxic coordinates of the injection site were −4.4 mm anteroposterior, −1.3 mm lateral, and -7.8 mm dorsoventral, according to the stereotaxic atlas of Paxinos and Watson^68^. After completion of the injection, the needle was gradually withdrawn over the course of 5 min, and the wound was closed using 4-0 nylon sutures. Animals were kept warm and allowed to recover from anaesthesia.

In this work, we aimed to investigate the preventive effects on Parkinson’s disease progression. We thus employed animals 3 weeks after 6-OHDA treatment, which is a week before the time when the degeneration of dopaminergic neurons induced by the neurotoxin is stabilised^24^. The animals were tested for their rotational responses to an intraperitoneal injection of R-apomorphine hydrochloride (0.5 mg/kg). Animals with>300 contralateral turns per h were selected as animals at the initial phase of Parkinson’s disease^69^, providing a 70% yield for model development.

#### Pump implantation

To prepare the animal groups treated with Alzet pumps, the tip of a 28-gauge brain infusion cannula (Alzet brain infusion kit; Durect, CA, USA) was fixed at the location between the striatum and substantia nigra (Supplementary Figs. S3 and S4). The pump body was inserted in a subcutaneous pocket prepared at the cervical upper part of the dorsum. As the catheter was connected between the cannula and pump body, the loaded solution could be continuously infused into the substantia nigra by the osmotic pressure that developed in the pump body by absorption of bodily fluids. During recovery, we followed the Rodent Anaesthesia guidelines and provided analgesia as indicated in the approved protocol.

#### Behavioural assessment

Rotational behaviour was assessed at 4, 5, 6, and 7 weeks after 6-OHDA MFB lesioning (i.e. 1, 2, 3, and 4 weeks after CoQ10 treatment, respectively). Rats were injected intraperitoneally with R-apomorphine hydrochloride (0.5 mg/kg) dissolved in 0.1% ascorbate-saline. Rotational responses were then immediately measured by placing rats in a white hemispheric plastic rotation bowl (42 cm in width at the top and 22 cm in depth). The number of rotations in ipsilateral and contralateral directions was recorded and expressed as net turns per h (i.e. the number of contralateral turns subtracted by the number of ipsilateral turns).

#### Brain tissue analysis

To further assess the effects of intrastriatally delivered CoQ10, we biopsied brain tissues from animals at the experimental end point (7 weeks after 6-OHDA injection). Rats were deeply anaesthetised via an intraperitoneal injection of a cocktail of xylazine (5 mg/kg) and zoletil 50 (20 mg/kg), followed by transcardial perfusion with approximately 50 mL of 0.9% NaCl solution with heparin. After perfusion with approximately 50 mL of ice-cold 4% paraformaldehyde, brains were removed and placed in a 50-mL conical tube containing ice-cold 4% paraformaldehyde for 1 day. Brains were then fully immersed daily in the order of 10%, 20%, and 30% sucrose solutions, respectively. The entire brain was then cut into 30-μm thick coronal sections for staining, using a cryostat (Leica CM 3000; Leica, Solms, Germany).

#### Tyrosine hydroxylase immunostaining

Every fifth section in the striatum and substantia nigra was collected for tyrosine hydroxylase immunohistochemistry using anti-tyrosine hydroxylase antibody. Tyrosine hydroxylase is an enzyme that converts the amino acid L-tyrosine into L-dopa, a precursor of dopamine; hence, the expression levels of tyrosine hydroxylase represent those of L-dopa and dopamine^70,71^. Briefly, slides with brain sections were washed with distilled water followed by PBS (three 5-min washes each). Then, endogenous peroxidase was quenched with 0.3% H_2_O_2_ for 30 min. Non-specific binding was blocked with 1.5% normal goat serum for 30 min (Vectastain mouse IgG ABC kit, Vector Laboratories, CA, USA). Slides were then incubated overnight at 4 °C with mouse polyclonal anti-tyrosine hydroxylase antibody (EMD Millopore MAB5280). Slides were then incubated with biotinylated secondary antibody for 60 min (Vectastain mouse IgG ABC kit), followed by application of horseradish peroxidase conjugate (Vectastain mouse IgG ABC kit) for 60 min. Then, 3,3′-diaminobenzidine mixed with distilled water, pH 7.5 buffered saline, and H_2_O_2_ were applied until the staining was determined to be optimal. To quantify the degree of tyrosine hydroxylase expression, an image from each slide was obtained at x100 magnification, which was assessed using image analysis software (Image Pro Plus; Media Cybernetics, MD, USA), following a previously reported protocol^72–74^.

#### Immunofluorescence staining

To observe the effects of CoQ10, coronal sections between the anterior and posterior biopsied brain were first prepared into slides, which were processed for immunofluorescence staining. Thus, nestin and GFAP-δ were utilised to evaluate neurogenesis, laminin was employed to assess angiogenesis, and TNF-α was used to examine neuroinflammation^42,43^. Briefly, slides were washed with PBS and permeabilised with PBS containing 0.05% v/v saponin and 5% v/v normal goat serum for 30 min. Then, non-specific binding was blocked with 1.5% normal goat serum for 30 min. For each staining, slides were first labelled with mouse anti-nestin (1:400; Millipore), rabbit anti-GFAP-δ (1:1000; Millipore), rabbit anti-laminin (1:400; Sigma), or rabbit anti-TNF-α (1:1000; Abcam) primary antibodies, respectively. Slides were incubated with the corresponding primary antibody overnight at 4 °C and subsequently treated with a fluorescence-labelled (Alexa 488 or 594) secondary antibody (1:500) for 1 h at 22 ± 2 °C. The slide was then washed and treated with a Vectashield mounting medium containing DAPI (Vector Laboratories, Burlingame, CA, USA). Immunostained slides were analysed on a confocal microscope (LSM 510; Carl Zeiss MicroImaging, Jena, Germany). To quantify the number of cells expressing laminin, GFAP-δ, nestin, or TNF-α in the striatum, three distinct slides were obtained from the anterior to posterior striatum at 150 μm intervals in each animal, respectively. From each slide, five images at x100 magnification were obtained without overlaps, where the threshold and acquisition parameters for each fluorescence signal were established, following the manufacturer’s setting of the microscope without changes. The image was then assessed using image analysis software (Image Pro Plus; Media Cybernetics, MD, USA). The cells with double positive staining on DAPI and an antibody of interest were counted and their number was expressed in percentage based on the total number of DAPI-positive cells^42^.

### Statistical analysis

The numbers of rotations in the behavioural test were analysed for statistical significance using an analysis of variance with α = 0.05, followed by pairwise comparisons using Tukey’s post-hoc test. Statistical analyses of immunofluorescence data were performed using Student’s *t*-tests. For all statistical analyses, differences were considered significant at p < 0.05.

## Supplementary information

Supplementary Information.

Supplementary Video S1.

## Data Availability

The data that support the findings of this study are available from the corresponding author, upon reasonable request.
