# Supplementary material for: Intrastriatal administration of coenzyme Q10 enhances neuroprotection in a Parkinson’s disease rat model
Source: Sci Rep. 2020 Jun 12;10:9572. doi: 10.1038/s41598-020-66493-w (PMC7293316; doi:10.1038/s41598-020-66493-w)
Supplement: Supplementary file 1 — Supplementary Information. [file 41598_2020_66493_MOESM1_ESM.docx]

**Supplementary Information**

**Intrastriatal administration of coenzyme Q10 enhances neuroprotection in a Parkinson’s disease rat model**

Hyung Woo Park^1, 2, 3, †^, Chun Gwon Park^4, †^, Min Park^5, †^, Seung Ho Lee^6^, Hye Ran Park^1, 2, 3^, Jaesung Lim^4^, Sun Ha Paek^*, 1, 2, 3^, and Young Bin Choy^*, 5, 6, 7^

^1^Department of Neurosurgery, Seoul National University College of Medicine, Seoul, 03080, Republic of Korea.

^2^Cancer Research Institute, Seoul National University College of Medicine, Seoul, 03080, Republic of Korea.

^3^Ischemic/Hypoxic Disease Institute, Seoul National University College of Medicine, Seoul, 03080, Republic of Korea.

^4^Department of Biomedical Engineering, SKKU Institute for Convergence, Sungkyunkwan University (SKKU), Suwon, 16419, Republic of Korea.

^5^Interdisciplinary Program in Bioengineering, College of Engineering, Seoul National University, Seoul, 08826, Republic of Korea.

^6^Institute of Medical & Biological Engineering, Medical Research Center, Seoul National University, Seoul, 03080, Republic of Korea.

^7^Department of Biomedical Engineering, Seoul National University College of Medicine, Seoul, 03080, Republic of Korea.

^†^These authors contributed equally as first authors to this work.

**^*^Corresponding Author (Prof. Sun Ha Paek)**

Department of Neurosurgery, Seoul National University College of Medicine, Seoul, 03080, Republic of Korea

E-mail: paeksh@snu.ac.kr

Tel: +82-2-2072-3993

Fax: +82-2-744-8459

**^*^Corresponding Author (Prof. Young Bin Choy)**

E-mail: ybchoy@snu.ac.kr

Tel: +82-2-740-8592

Fax: +82-2-741-6303

Department of Biomedical Engineering, Seoul National University College of Medicine, Seoul, 03080, Republic of Korea


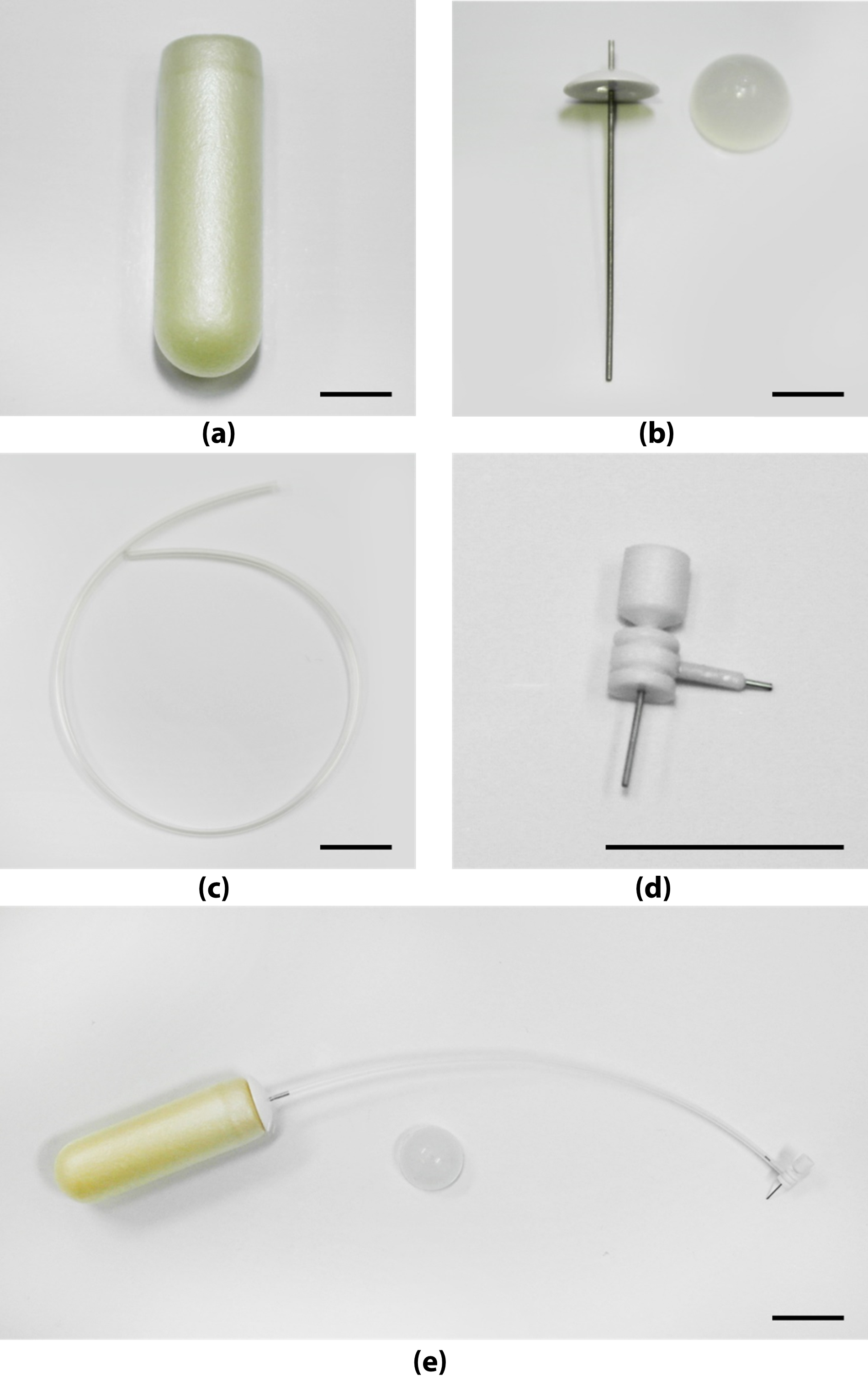


**Supplementary Figure S1. Images of the Alzet osmotic pump.** The pump includes (a) the body for drug reservoir and osmotic pumping, (b) flow moderator, (c) catheter tube, and (d) infusion kit cannula. (E) The parts are assembled to produce a full system. The total displaced volume and weight of the Alzet pump used in this study were 6.5 mL and 5.1 g, respectively. The scale bars are 1 cm.


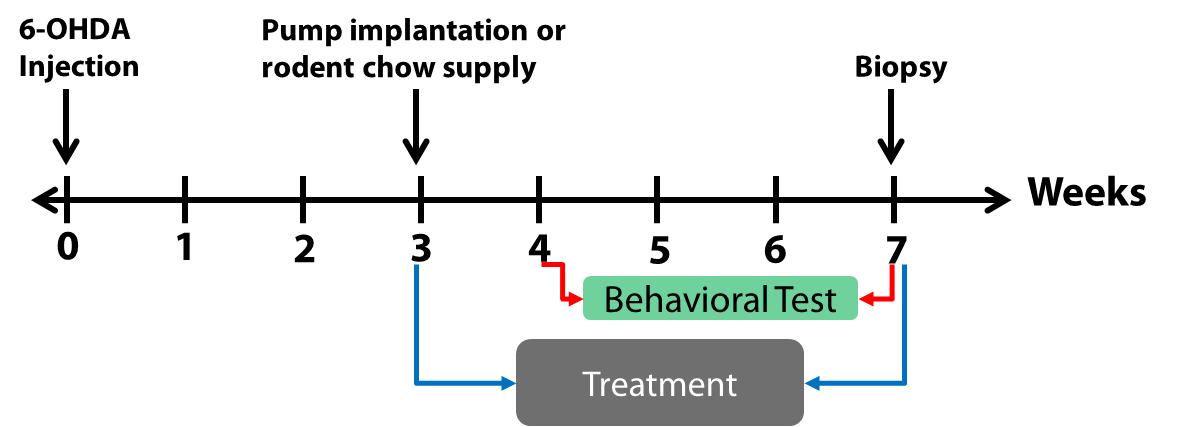


**Supplementary Figure S2. Experimental design with Parkinson’s disease animal models.** The treatment, i.e. pump implantation or supply of rodent chow containing CoQ10, started 3 weeks after 6-OHDA injection. Behavioural tests were performed at 4–7 weeks and the brains were biopsied for immunochemistry and immunofluorescence analyses at the end point of experiments (7 weeks).


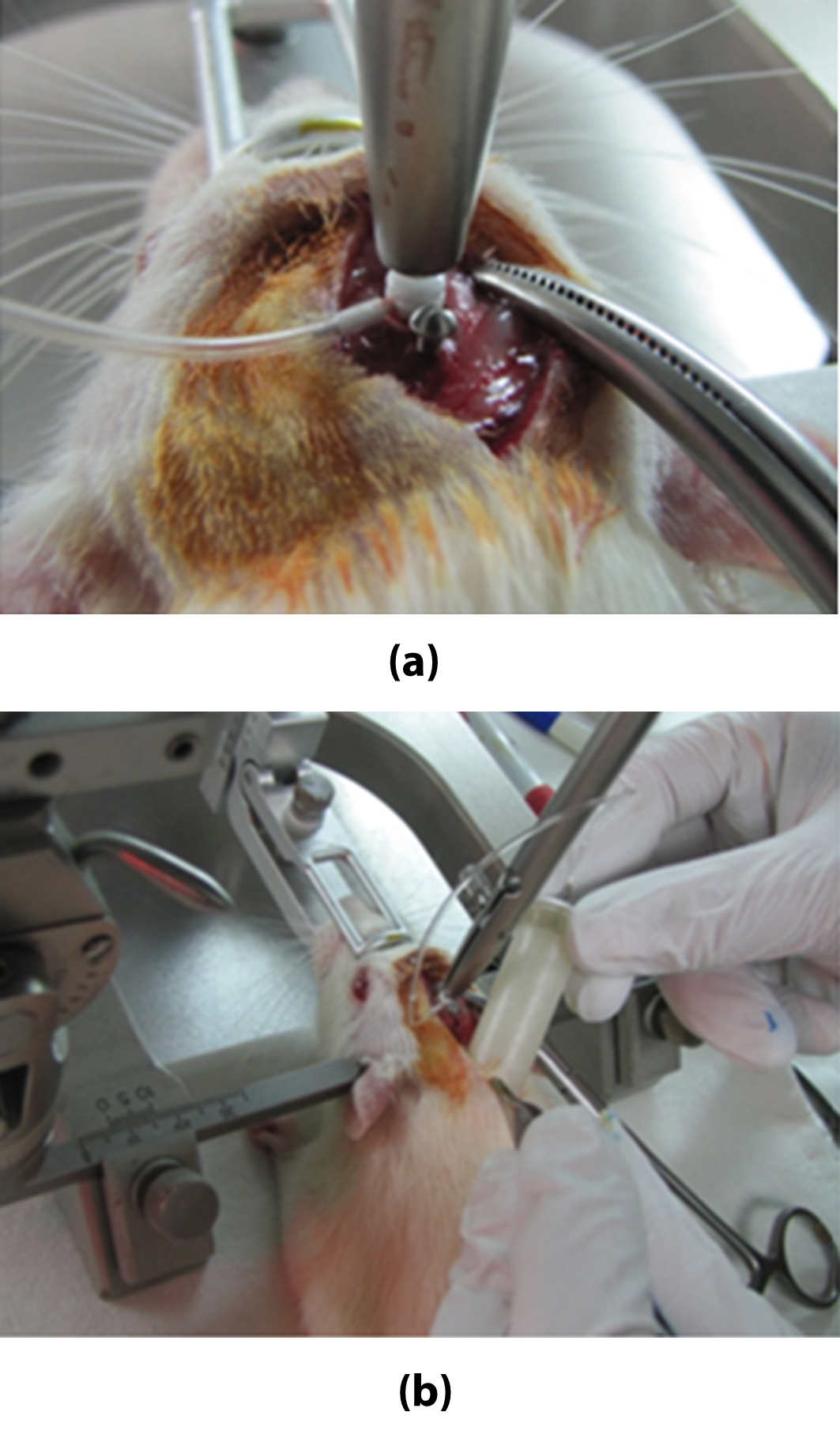


**Supplementary Figure S3. *In vivo* pump implantation procedures.** (a) The cannula of the pump was fixed to the target area in the brain. (b) The pump body was implanted into the subcutaneous space at the back of the animal with a catheter connected to the body and cannula.


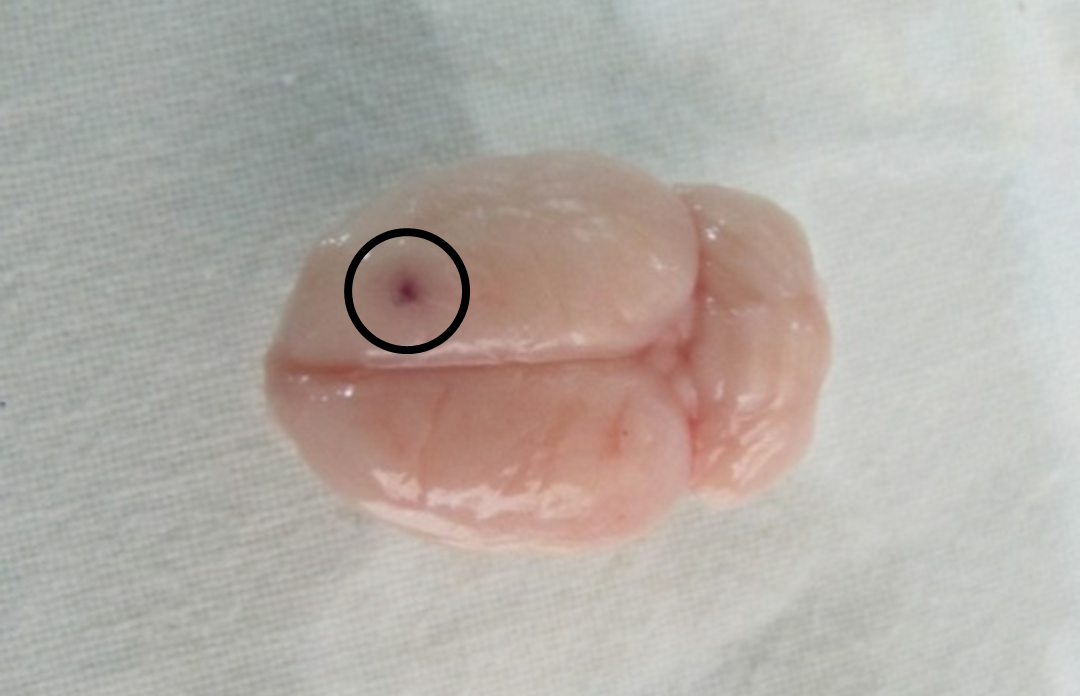


**Supplementary Figure S4. Biopsied brain showing the site of catheter insertion.**


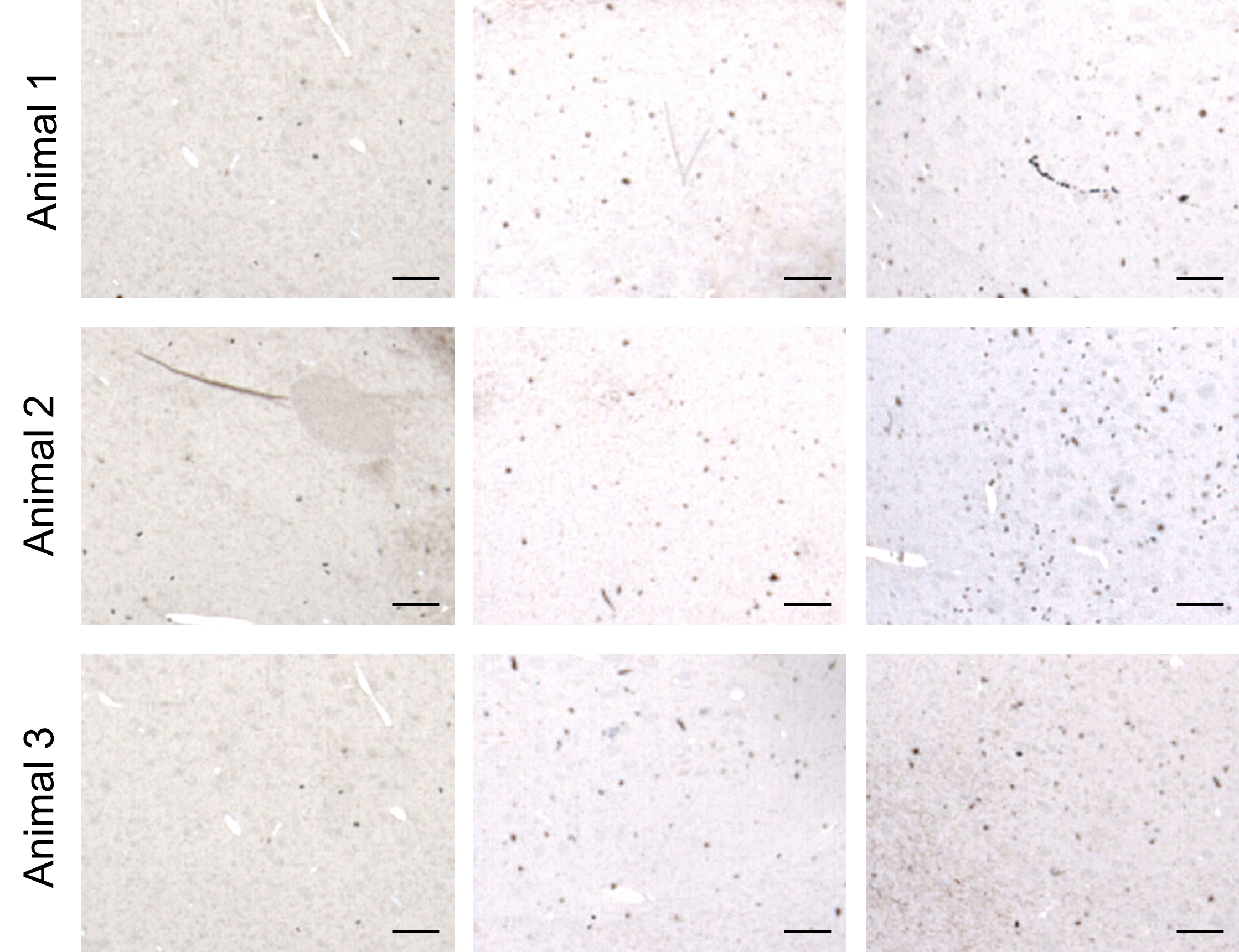


(a)


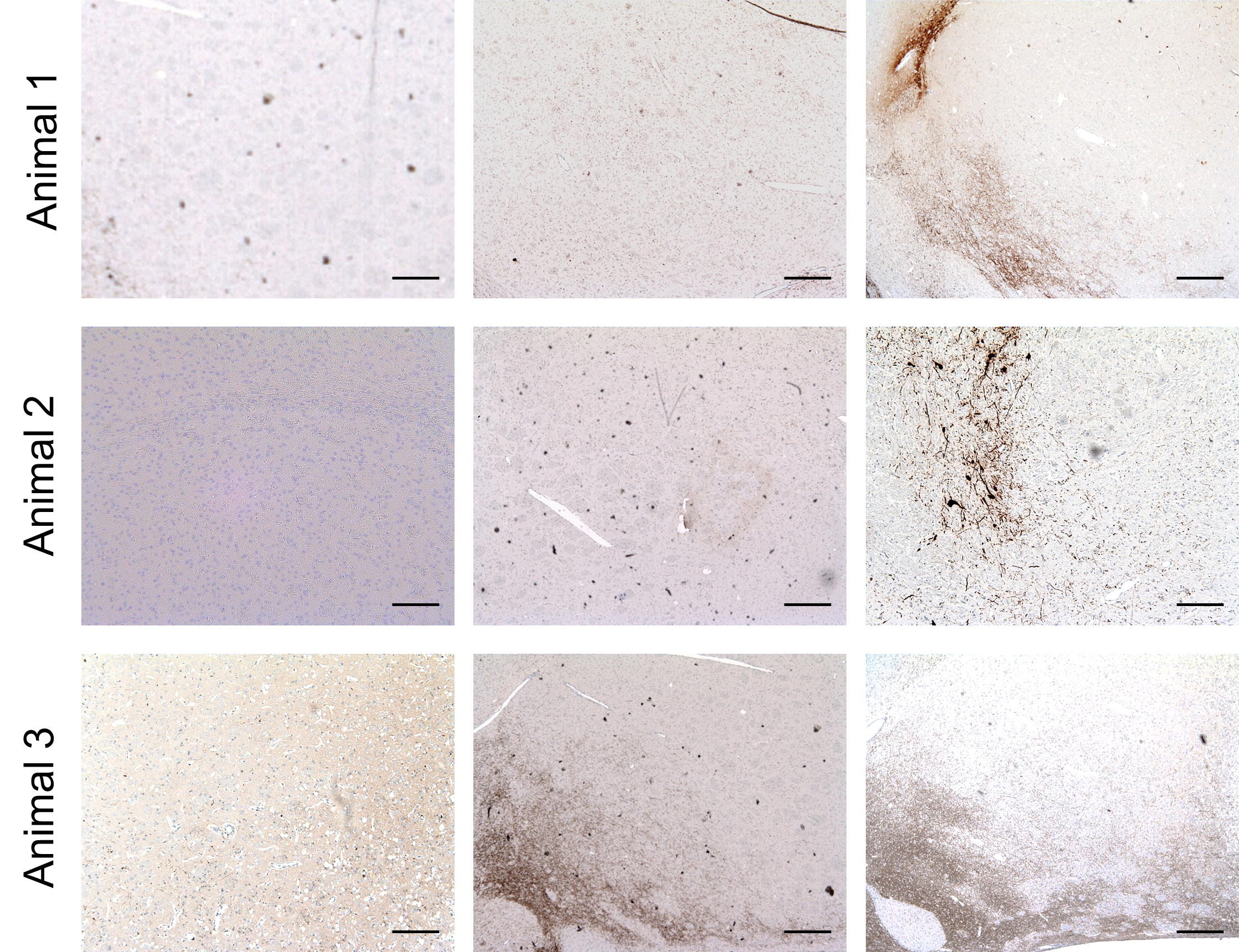
 (b)


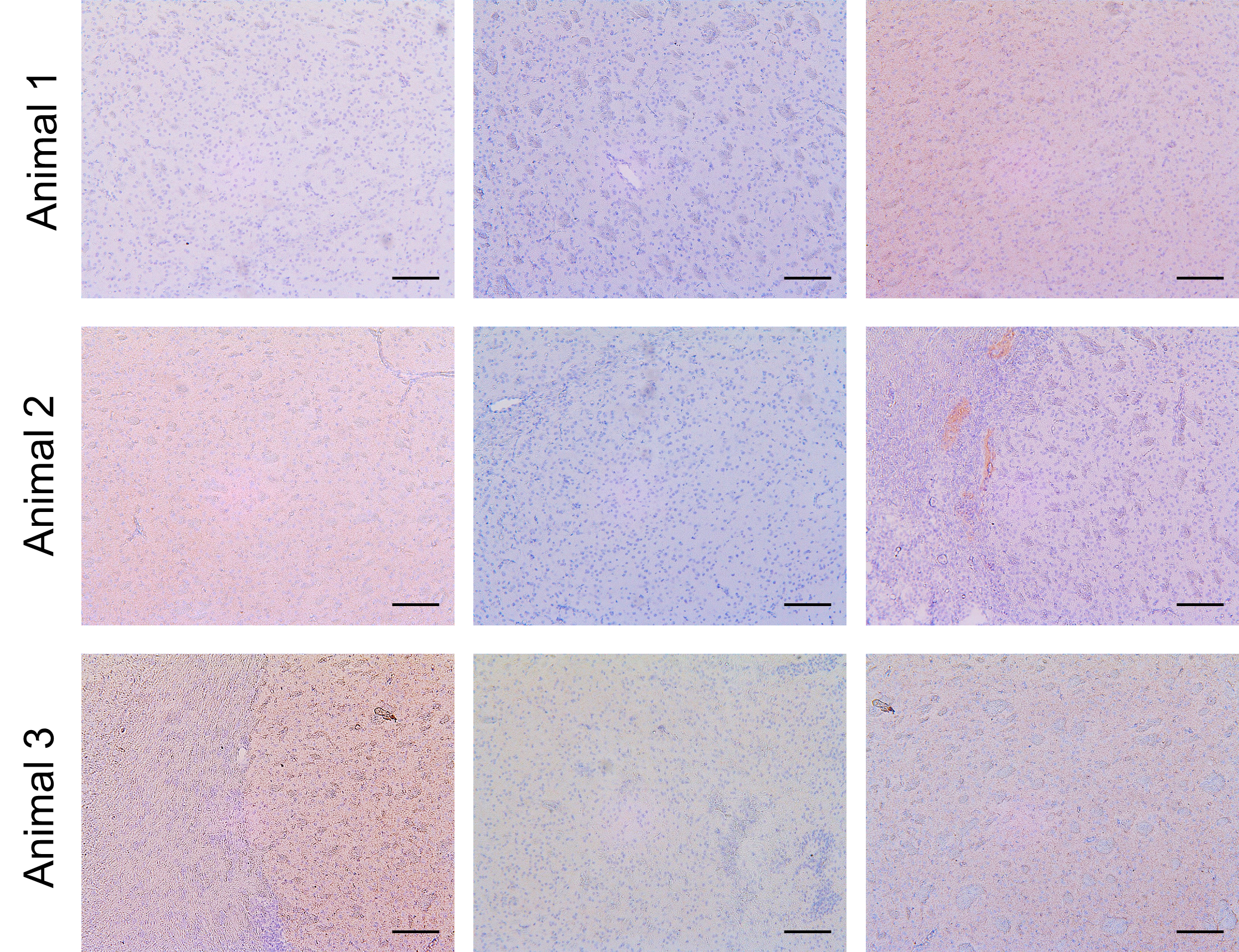


(c)


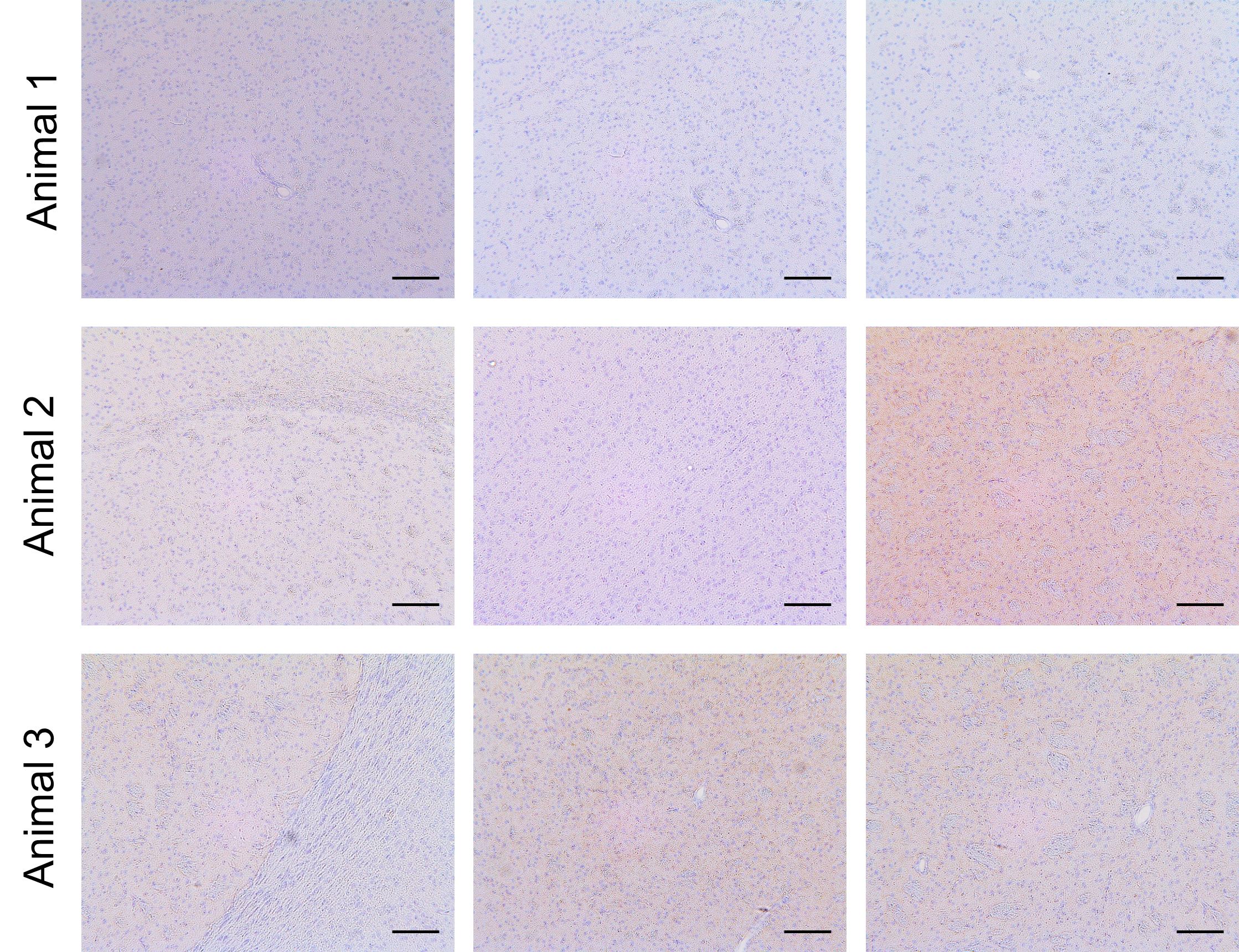


(d)


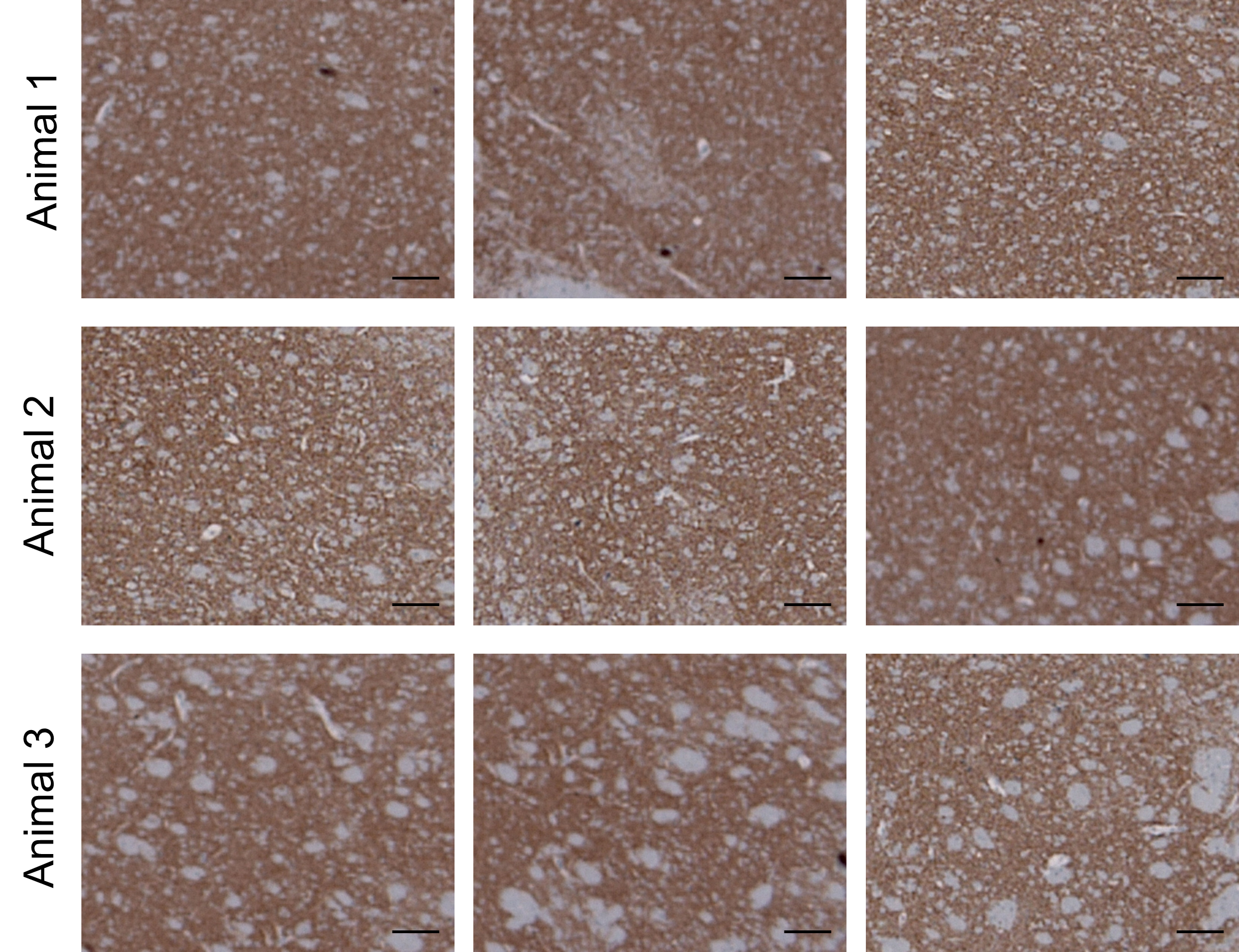


(e)

**Supplementary Figure S5. Tyrosine hydroxylase expression in the lesioned (right) striatum.** Three distinct images were obtained from each of all animals in the (a) no treatment group, (b) oral CoQ10 group, (c) Alzet-PBS group, (d) Alzet-low CoQ10 group, and (e) Alzet-high CoQ10 group. The scale bars are 100 µm.


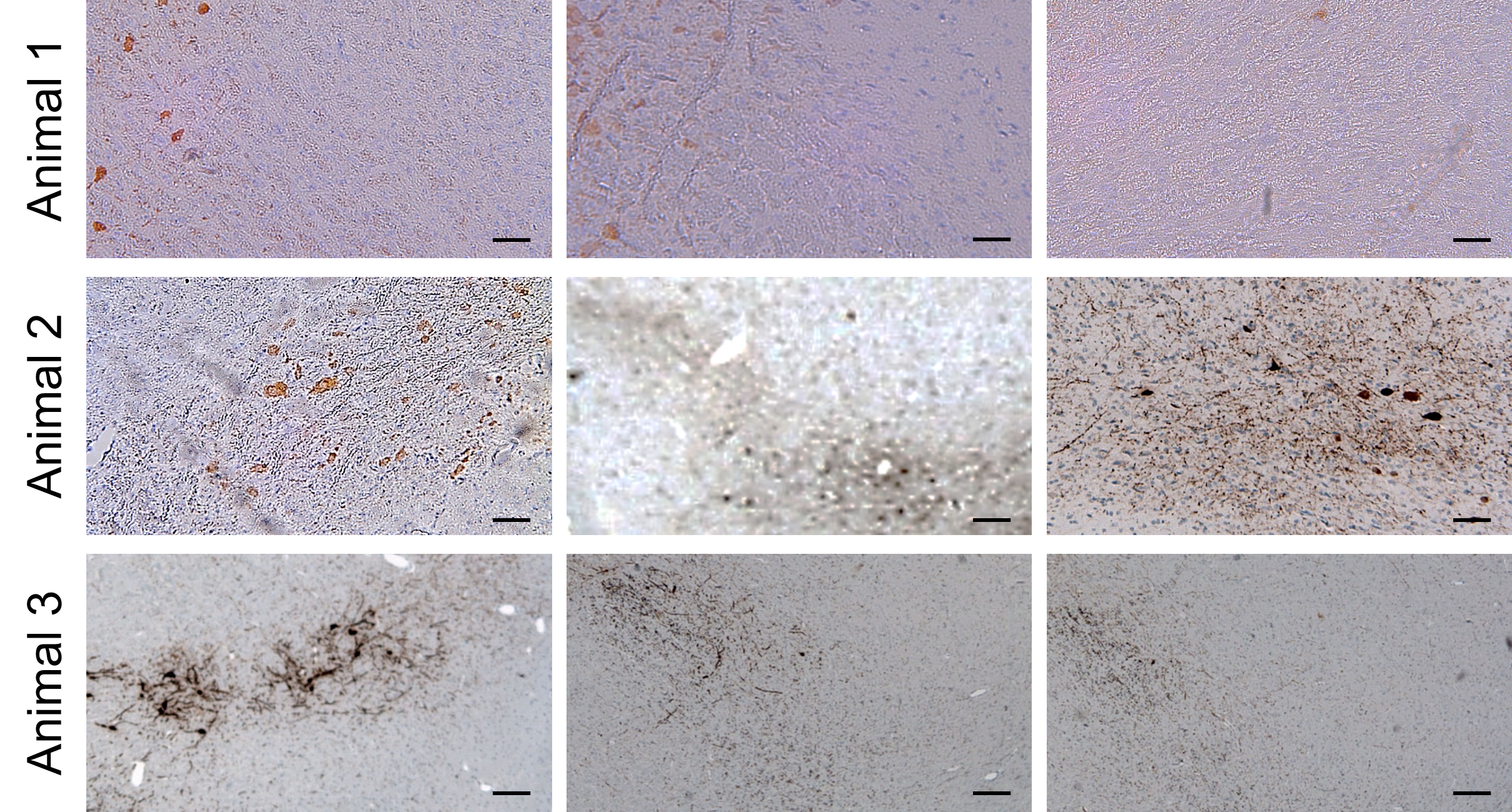


(a)


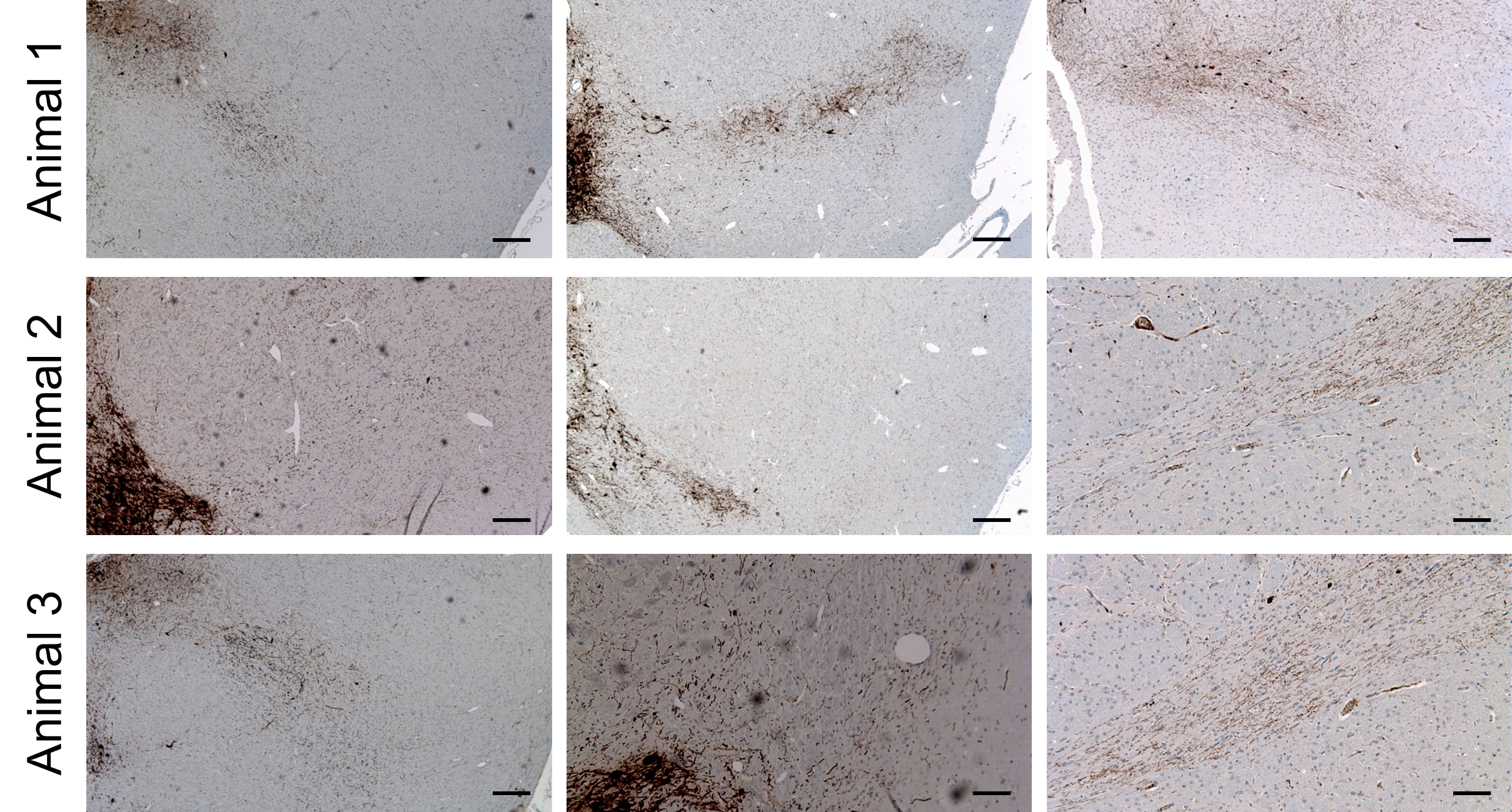


(b)


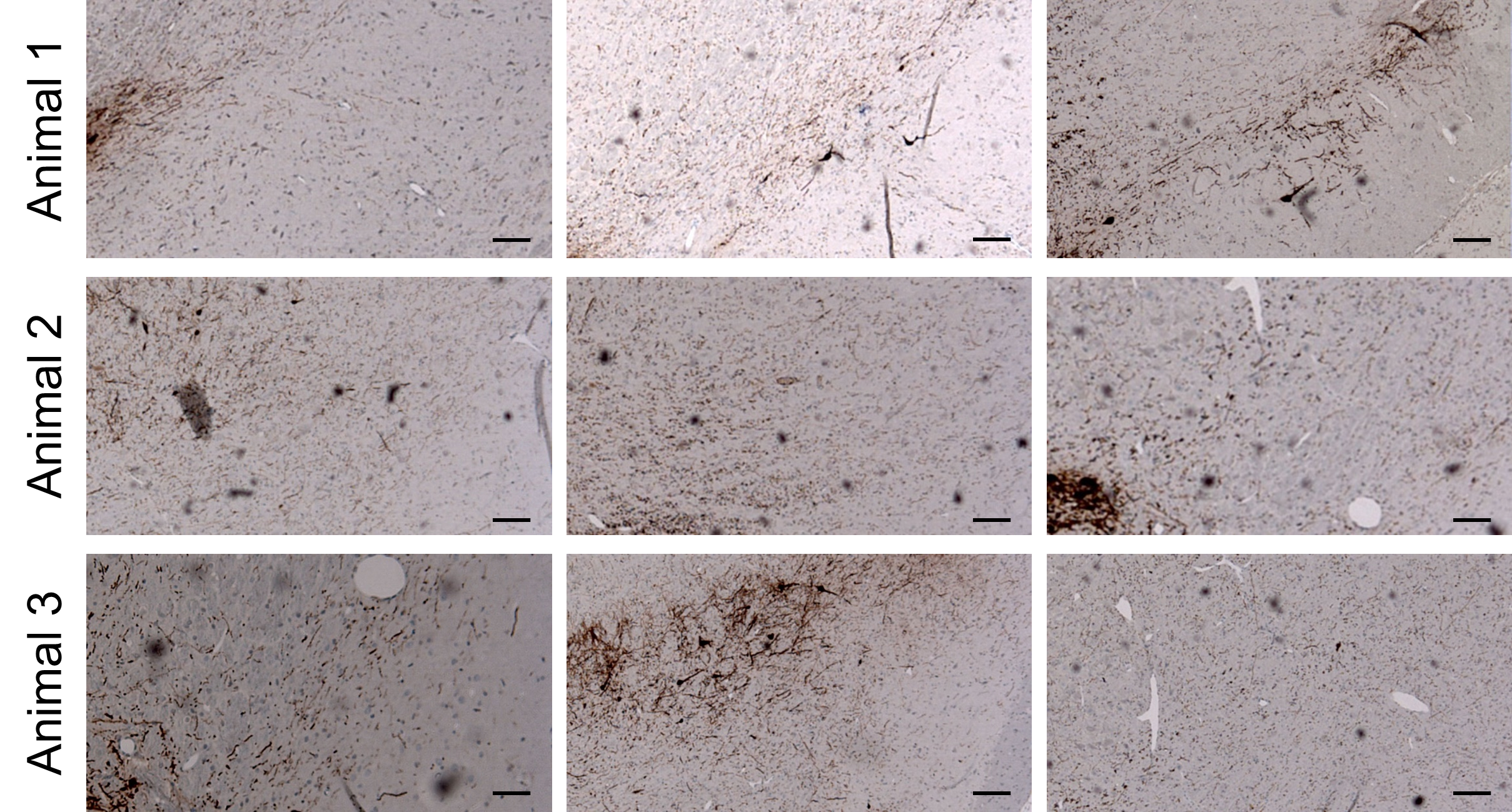


(c)


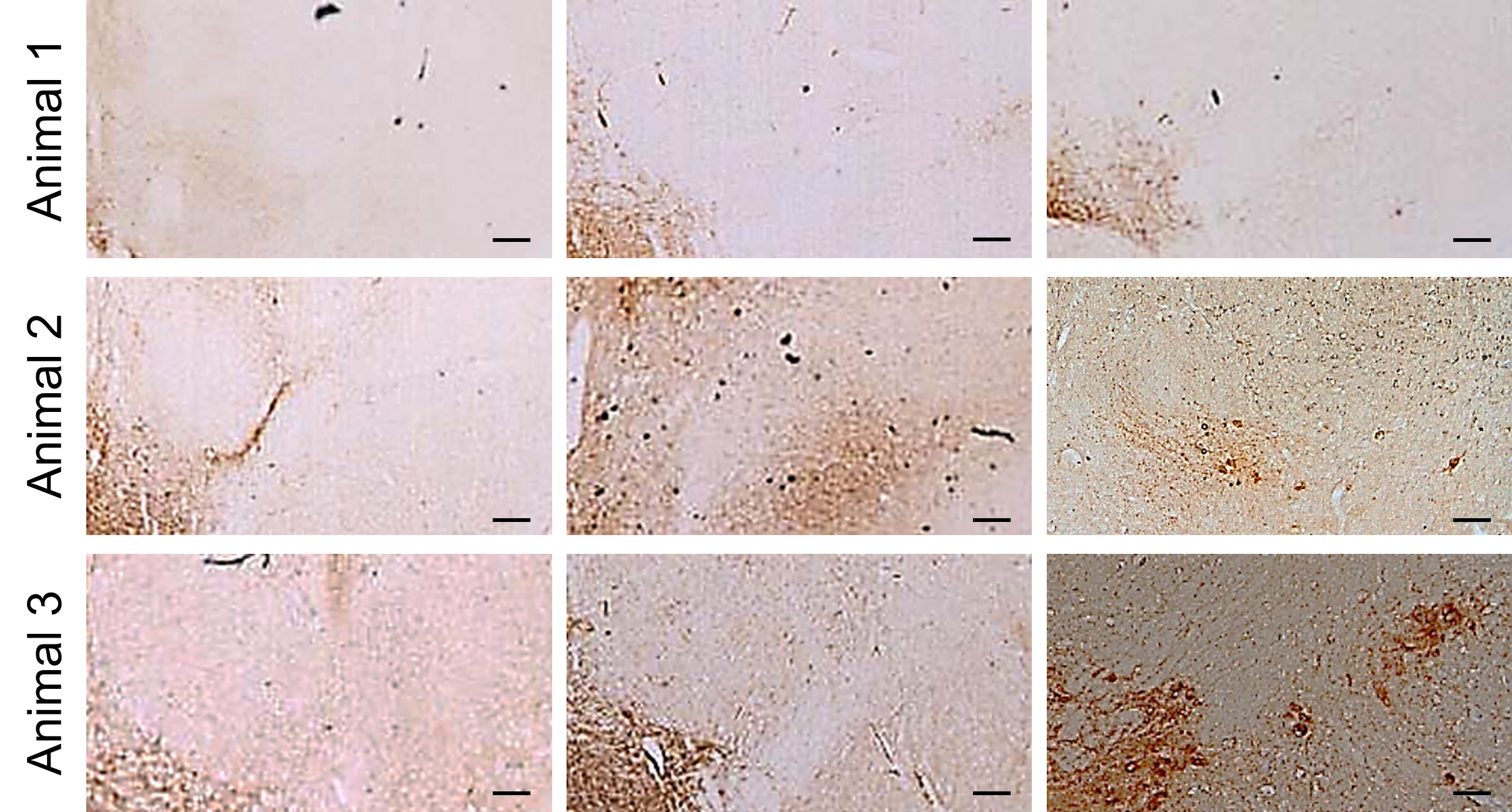


(d)


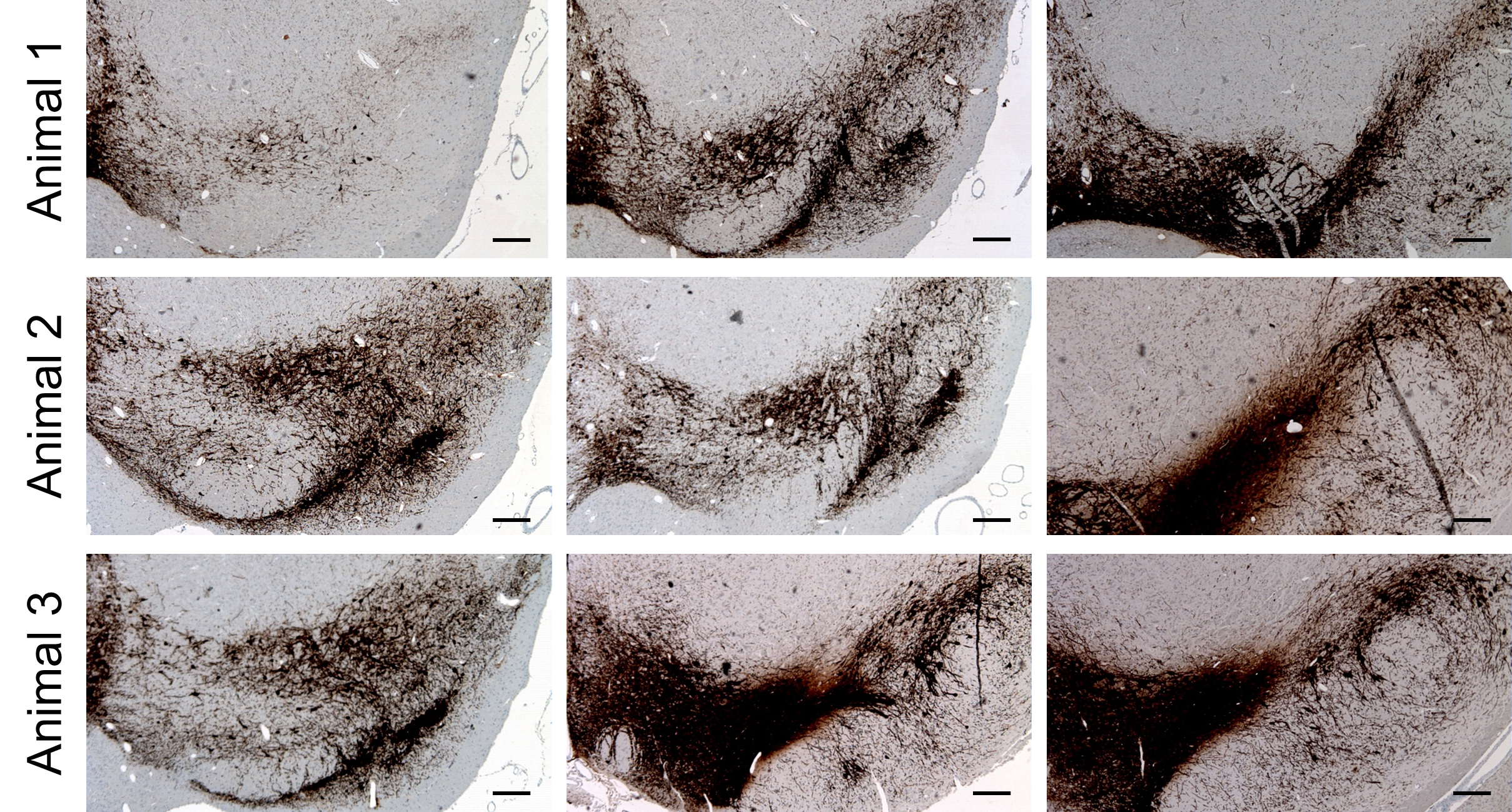


(e)

**Supplementary Figure S6. Tyrosine hydroxylase expression in the lesioned (right) substantia nigra.** Three distinct images were obtained from each of all animals in the (a) no treatment group, (b) oral CoQ10 group, (c) Alzet-PBS group, (d) Alzet-low CoQ10 group, and (e) Alzet-high CoQ10 group. The scale bars are 100 µm.


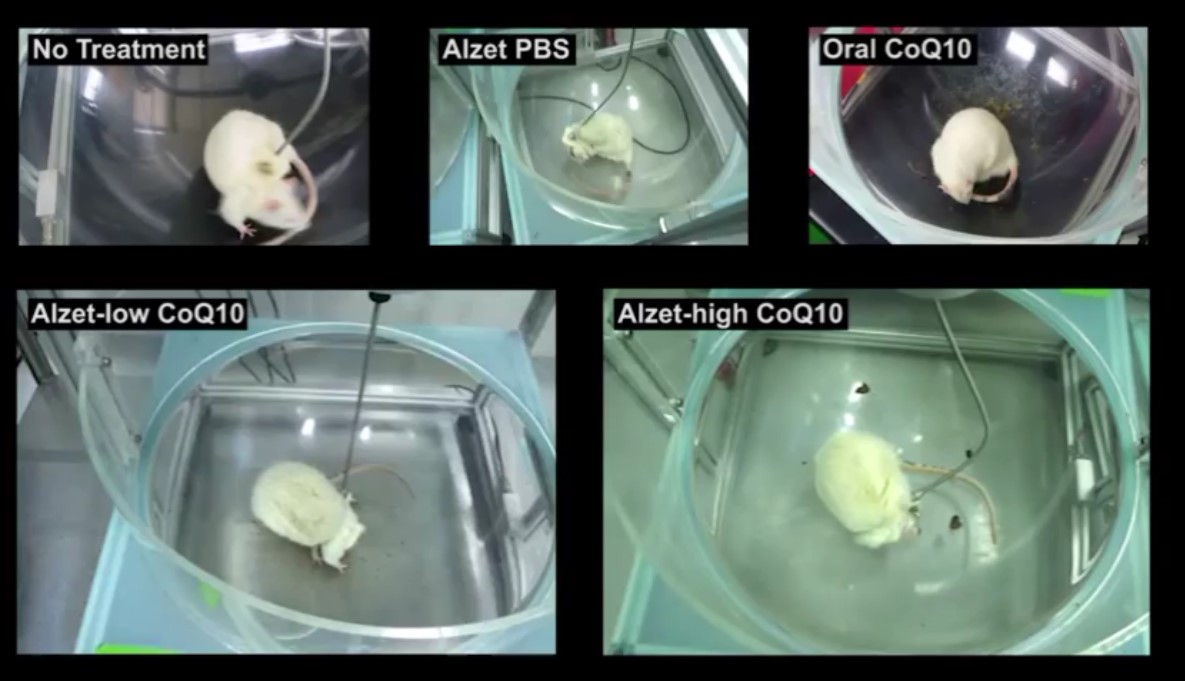


**Supplementary Video S1.** **Rotational behaviours from the differently treated animal groups.**
